# Predicting the evolutionary and structural compensation in Tat ARM region across HIV-1 groups using machine learning approach

**DOI:** 10.1038/s41598-026-49324-2

**Published:** 2026-04-24

**Authors:** Ridwanul Karim, Md Sakil Arman, K. M. Kamrul Hasan, Zafrul Hasan

**Affiliations:** Department of Biochemistry and Molecular Biology, Shahjalal University of Science and Technology, Sylhet, 3114 Bangladesh

**Keywords:** HIV-1 Tat, TAR RNA, dn/ds, co-evolution, codon-codon covariation, Phylogenetic dependency network, Arginine Rich Motif, Compensatory mutation, Machine learning, Computational biology and bioinformatics, Structural biology

## Abstract

Trans-activator of transcription (Tat), a key regulatory protein of HIV-1, binds the TAR RNA element and is essential for early viral pathogenesis. Remarkably, Tat from HIV-1 groups M, N, O, and P shows notable sequence variability, especially in the critical Arginine-Rich Motifs (ARM) required for TAR binding, presenting both challenges and opportunities for therapeutic targeting. Computational analysis and machine learning (ML) approaches were utilized to analyze over 45,000 HIV-1 *tat* sequences, retrieved from the Los Alamos National Laboratory (LANL) database spanning 1997 to 2021. Host-mediated selection on *tat* gene ensued distinct group-specific pattern, reflected in non-synonymous vs. synonymous ratio (dn/ds) of 2.20, 2.04, 2.45, and 1.08 for groups M, N, O, and P, with corresponding entropy scores of 0.21, 0.35, 0.14, and 0.01, respectively. Groups M and N exhibited comparable selective pressure, while O and P evolved under distinct environments. Remarkably, group-specific sequence variability was evident in TAR binding region, particularly within the ARM, likely influencing viral pathogenicity. Docking analysis of wild-type Tat-TAR complexes supported this, showing binding affinities (docking scores) of − 313, − 242, − 282, and − 239, for groups M, N, O, and P, respectively. Phylogenetic Dependency Network (PDN) analysis revealed 1375, 36, 135, and 6 co-evolutionary associations within Tat for these groups, including 183, 1, 12, and 0 from the ARM motif. Structural modeling combined with PDN analysis identified 355 associated mutations in group M conferring structural compensation, of which 40 were from the ARM region. By leveraging these structural and co-evolutionary insights, we developed an ML model to classify compensatory mutations. HIV-1 Tat evolves differentially under host-mediated selective pressures, with group M exhibiting the highest adaptability through coevolutionary compensation, reflecting its global infection dynamics. Targeting conserved regions and compensatory mutational pathways in Tat may yield novel strategies for HIV-1 therapeutics.

**Supplementary Information:**

The online version contains supplementary material available at 10.1038/s41598-026-49324-2.

## Introduction

Global Human Immunodeficiency Virus type-1 (HIV-1) strains are classified into four groups: M (Major), N (non-M/non-O), O (Outlier), and P (Pending or Putative), of which group M accounts for around 90–95% of global HIV infections^1^. During viral pathogenesis, HIV-1 encounters host-imposed selective pressures, including cellular immune responses (CD8+, CD4+, B-cells, and cytokines), antiretroviral therapy, and host restriction factors. Combined with its high replication rate and error-prone reverse transcriptase, these forces shape its evolutionary dynamics and mutational plasticity to evade host-mediated pressure^2^. Host immune–mediated escape mutations enable HIV-1 to evade immune recognition, often inducing several primary mutations in the process^3,4^. However, immune-escape mutations, particularly missense ones, are often detrimental to the virus, necessitating secondary mutations at nearby or distant codons to counteract the deleterious effects of the primary mutation and restore viral fitness (e.g., replication efficiency)^5^. Interestingly, the emergence of these secondary compensatory mutations can be predicted through data-driven analyses using contemporary bioinformatics and machine-learning approaches^6–9^.

Among the two regulatory proteins of HIV-1, the Trans-activator of transcription (Tat) is essential for viral gene expression, and its high sequence variability suggests strong host-mediated selective pressure^10^. Tat binds to a specific RNA sequence called the trans-activation response (TAR) element at the 5′ end of viral transcripts^11,12^. It recruits host transcription factors, such as P-TEFb, along with RNA polymerase II, thereby initiating viral transcription and enhancing early pathogenesis^13–15^. While functionally active as a transcriptional regulator, Tat also exhibits high adaptability within the host environment, enabling its effectiveness in viral pathogenesis under host selection pressure^16^. Multiple studies have reported sequence polymorphisms in Tat among the clades of HIV-1 group M. Notably, single mutations at positions 57 and 63 in subtype C have been shown to enhance transcriptional activity compared to subtypes B and E in primary T-cell lines^16,17^. This suggests that Tat sequence polymorphisms significantly influence viral replication, potentially explaining the greater structural adaptability and pathogenicity of group M compared to groups N, O, and P.

The HIV-1 Tat protein consists of approximately 101 amino acids and comprises six domains translated from two separate exons^16^. Tat exon 1 encodes proline-rich, cysteine-rich, core, basic, and glutamine-rich domains (residues 1–72), which together are sufficient for transactivation of the HIV-1 LTR^14–17^. In contrast, Tat exon 2 (residues 73–101) contributes to protein stability, nuclear localization, and interactions with host factors. Remarkably, the well-conserved arginine-rich motif (^49^RKKRRQRRR^57^) in exon 1 is crucial for interaction with the TAR element^18–20^. Mutations within ARM, predominantly residues 49 to 53, have been shown to disrupt TAR binding and LTR transactivation^21–27^. These alterations often lead to reduced RNA-binding efficiency, impaired nuclear import, and eventually compromised transcriptional activation. A recent study has shown that the ARM of Tat suppresses dicer-dependent RNA interference (RNAi) beyond its classical role^28^. Notably, the ARM is being explored in broader efforts to modulate Tat’s role in HIV-1 latency and transcription. For instance, synthetic ARM mimetics (e.g., Tat11) and small molecules that disrupt TAR binding or nuclear localization can impair Tat’s nuclear import and transcriptional activity^29,30^. This makes the ARM a promising druggable hotspot for both latency-reversing and latency-maintaining therapeutic strategies.

Despite these functional insights, most prior studies have focused on subtype-specific variation within group M or limited regional cohorts^31,32^. A comprehensive cross-group evolutionary comparison integrating structural constraints, compensatory adaptation, and co-evolutionary dynamics of Tat–TAR interaction remains lacking. Given that HIV-1 groups M, N, O, and P evolved independently following zoonotic transmission events, it remains unclear whether shared structural constraints are preserved across groups despite divergent evolutionary trajectories.

We hypothesized that although HIV-1 groups M, N, O, and P have undergone independent evolutionary diversification, conserved structural constraints within the Tat–TAR interaction interface are maintained through compensatory adaptation and co-evolving residue networks. To test this hypothesis, we performed a global-scale evolutionary and structural analysis of 25 years (1997–2021) of HIV-1 Tat sequences, retrieved from the Los Alamos National Laboratory (LANL) database. Using integrative computational approaches, which included, selection pressure analysis, entropy profiling, structural modeling, co-evolutionary network inference, and machine-learning–based classification, we aimed to (i) identify conserved and variable regions across groups, (ii) characterize compensatory mutational patterns within the Tat–TAR interface, and (iii) define structural resilience mechanisms that enable functional preservation under host-mediated selection pressure.

To design broadly effective HIV-1 therapeutics targeting the Tat ARM region, it is crucial to understand the commonalities and differences among major groups and clades in terms of sequence polymorphism, evolutionary dynamics, and compensatory mechanisms. Thus, this study utilizes 25 years of global HIV-1 sequence data (1997–2021) from the Los Alamos National Laboratory (LANL) database to predict structural resilience, adaptability, and compensatory patterns under host selection pressure through computational and machine-learning approaches. Therefore, our findings may inform future HIV-1 therapies by highlighting conserved and compensatory mutational pathways for targeted intervention.

## Result

### Selection pressure and amino acid variability of HIV-1 Tat

**Fig. 1:**
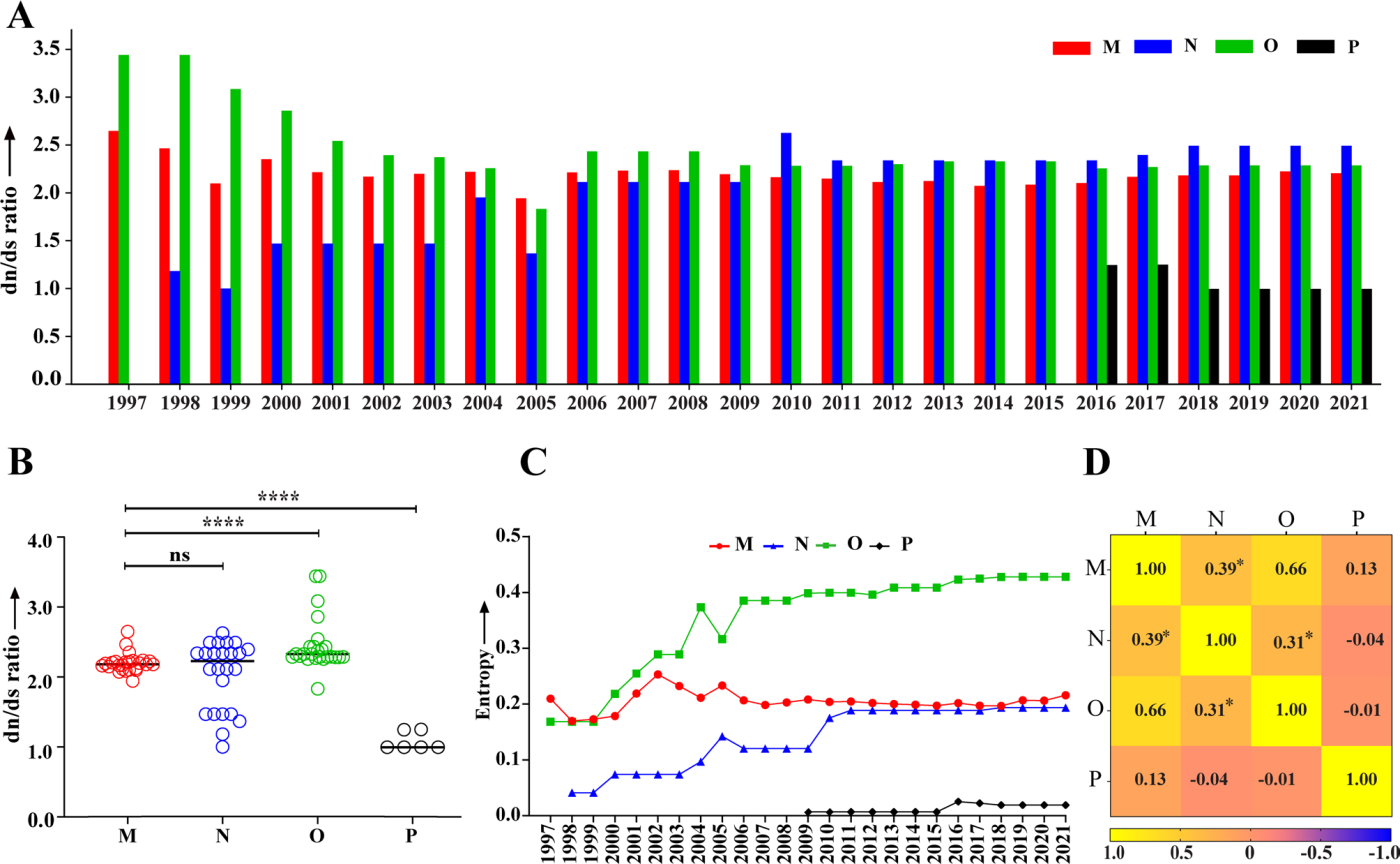
Comparative analysis of selection pressure among HIV-1 groups M, N, O, and P (**A**) Average dn/ds ratio among the HIV-1 groups, M (*n* = 43,087, red), O (*n* = 546, green), N (*n* = 160, blue), and P (*n* = 33, black) over time. (**B**) Mann-Whitney U test showing significant differences in temporal selection pressure between HIV-1 group M and groups O and P. (**C**) Average entropy scores of the four HIV-1 groups over time, representing the amino acid variability and diversity within each group. (**D**) Spearman correlation matrix showing pairwise correlations of entropy scores across HIV-1 groups M, N, O, and P; asterisks (*) indicate statistically significant correlations.

To infer the nature of selection pressure acting on the protein-coding gene of HIV-1 Tat, we calculated the nonsynonymous vs. synonymous substitution rate (dn/ds) using the *tat* gene sequences for 25 years (1997–2021) from groups M, N, O, and P. This analysis identified HIV-1 *tat* as being under adaptive selection, with distinct evolutionary dynamics observed among the groups (Fig. 1A). The overall selection pressure (dn/ds) for groups M, N, O, and P was 2.20, 2.04, 2.45, and 1.08, respectively. A notable fluctuation in the dn/ds ratio was observed over the years, except for group N, which showed a gradual decline (Fig. 1A). Interestingly, evolutionary pressures appeared to converge across all groups except P. These preliminary results indicate that the *tat* gene is undergoing strong positive selection (dn/ds ≥ 1.0) imposed by the human host globally. When comparing the less prevalent groups N, O, and P with the globally dominant group M, significant differences were observed between group M and groups O and P by the Mann-Whitney U test, suggesting a closer evolutionary similarity between groups M and N (Fig. 1B). To examine amino acid variability in the Tat protein under host selection pressure, we used Shannon entropy scores to assess residue diversity at each position across the intact open reading frame (ORF) in a cross-sectional manner^10^. While the average entropy scores were 0.21, 0.14, 0.35, and 0.01 for groups M, N, O, and P, respectively, group O showed higher variability, indicating greater adaptive capacity, whereas group M showed moderate variability, consistent with its widespread persistence. In contrast, groups N and P showed overall gradual increases in entropy, suggesting ongoing diversification in N and minimal variation in P, reflecting its rarity and limited geographic distribution (Fig. 1C). In addition, Spearman correlation matrix analysis also supports this evolutionary adaptability among the groups. Moderate corrections were observed for group M with O (0.66) and N (0.39), while low correlation with P (0.13), indicating divergent evolutionary routes (Fig. 1D). Notably, the evolutionary resemblance between M and N (Fig. 1D) may stem from shared host origin (Supplementary Fig. SF2). Importantly, to evaluate whether unequal sampling depth influenced these patterns, we performed a controlled down-sampling analysis. Although group M contained substantially more sequences, all evolutionary analyses were conducted separately within each group, preventing pooled numerical bias. Additionally, group M was randomly downsized to 739 sequences (by randomly selecting an equal number from the past 25 years) to match the combined sizes of the non-M groups (*N* = 160, O = 546, *P* = 33). Reanalysis of selection pressure and entropy with this balanced dataset indicated no sampling artifacts in lineage-specific dn/ds estimates, neither across the entire ORF nor within individual Tat domains (Supplementary Fig. SF1A–B). Phylogenetic reconstruction of the balanced dataset preserved distinct group-specific clustering (Supplementary Fig. SF1C), confirming that the adaptive signatures and variability patterns represent intrinsic evolutionary characteristics rather than consequences of sampling imbalance.

## Sequence conservation and structural variation of Tat ARM region across HIV-1 groups

**Fig. 2:**
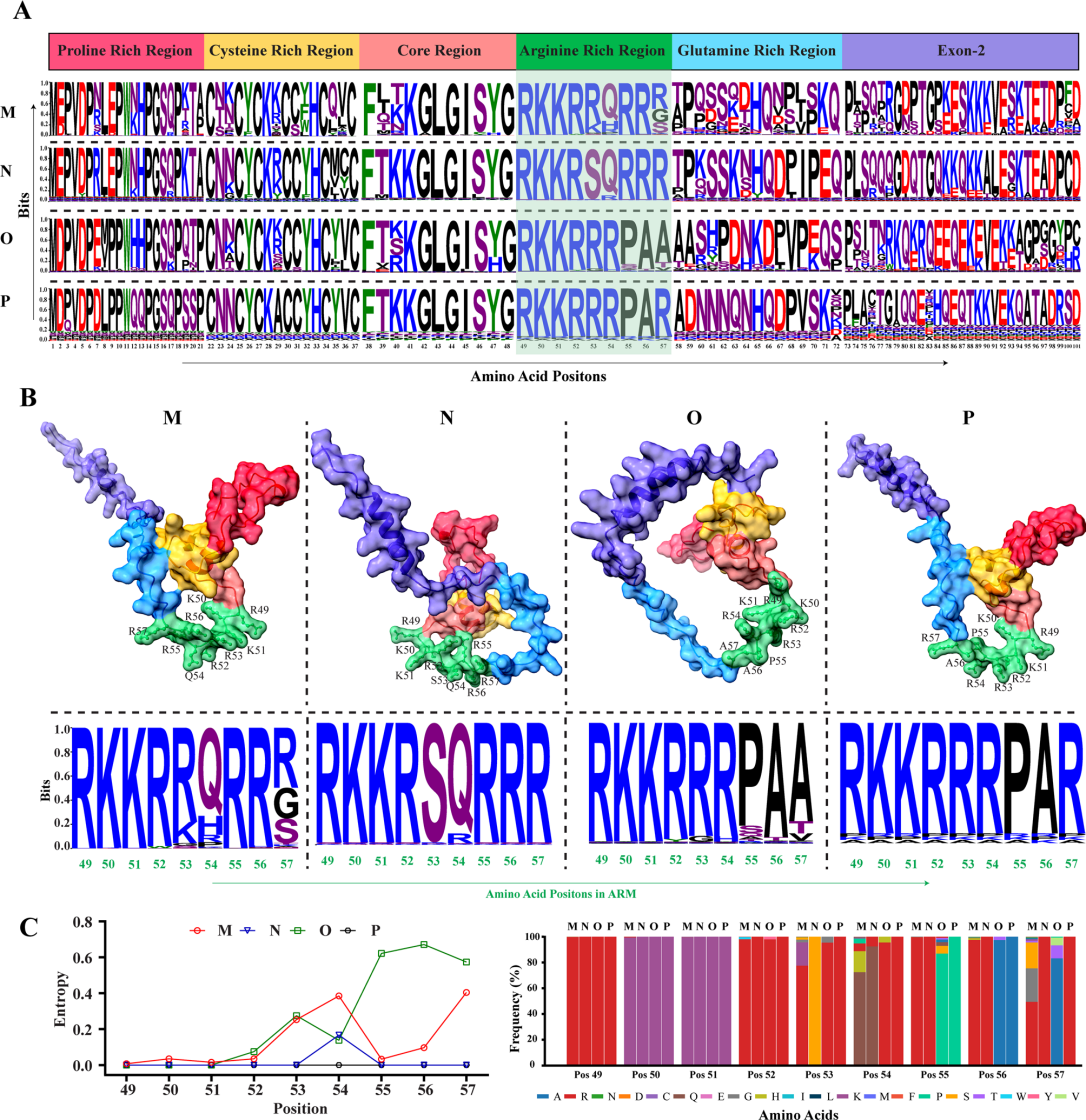
Sequence and structural comparison of HIV-1 Tat of groups M, N, O, and P. (**A**) Sequence logo of amino acid conservation and variability across various HIV-1 groups. (**B**) Three-dimensional structure of HIV-1 Tat proteins predicted by AlphaFold2, based on consensus sequences from each group (M: 43,087 sequences; O: 546; N: 160; P: 33). Motifs are color-coded: proline-rich (red), cysteine-rich (yellow), core (pink), arginine-rich (green), glutamine-rich (cyan), and exon-2 (purple). The arginine-rich motif (ARM), critical for TAR binding, is highlighted with ball-and-stick models of specific amino acids to emphasize structural variations. (**C**) The Shannon entropy per position (left panel) and the frequency (%) of observed amino acids at each position (right panel) of the ARM region among the groups.

To visualize sequence variability and conserved regions of the Tat protein among groups M, N, O, and P, we generated sequence logos from multiple sequence alignments of the HIV-1 Tat sequence dataset (*n* = 43,826, 1997–2021), illustrating amino acid variability and conservation across the open reading frame (Fig. 2A). Among the different motifs of the Tat protein, the glutamine-rich region (residues 58-72)  and C-terminal exon 2 (residues 73–101) showed the highest amino acid variability across HIV-1 groups, whereas the core region^ (residues 38-48)^, arginine-rich motif (residues 49-57), and N-terminal proline-rich region (residues 1-21)  were relatively conserved, indicating diverse selective pressures among Tat amino acid residues. To assess the impact of group-specific sequence variability on the conformation of unbound Tat protein, we predicted the 3D structure using AlphaFold2. Consensus reference sequences were generated by Geneious Prime (v2023.1.1) (http://www.geneious.com) from the multiple sequence alignments of each group, M (*n* = 43,087), N (*n* = 160), O (*n* = 546), and P (*n* = 33), and used as input for AlphaFold2 in Google Colab (Fig. 2B). Remarkably, notable conformational variations were observed across the groups, particularly within and around the ARM region (residues 49-57), suggesting that group-specific amino acid signatures may influence Tat’s structural conformation and consequently, its TAR-binding capacity (Fig. 2B, upper panel). This provides a plausible framework for interpreting the spatial and functional relationships among different regions or domains of Tat, emphasizing how amino acid variability at positions 53 to 57 may influence structural dynamics in other regions, such as the proline-rich (red), cysteine-rich (yellow), core (pink), glutamine-rich (cyan), and exon 2 (purple) domains (Fig. 2B, upper panel). The AlphaFold2-predicted full-length Tat models for HIV-1 M, N, O, and P showed comparable moderate confidence scores (average pLDDT: 70.02, 70.04, 70.38, and 71.48, respectively), consistent with the absence of an experimentally resolved full-length Tat structure and the intrinsic flexibility of regions. Focusing on the variability of the TAR RNA–binding region of Tat, we compared the arginine-rich motif (ARM) across HIV-1 groups. Positions 49 to 52 showed consistent amino acid conservation, whereas variability was observed from positions 53 to 57 across the groups (Fig. 2B, lower panel). In group M, arginine (R) and glutamine (Q) were predominant at positions 53 and 54, respectively; serine (S) and glutamine (Q) were common in group N, while arginine (R) occupied both positions in groups O and P. We also observed distinct signature patterns at positions 55 to 57, where groups M (⁵⁵RRR/G/S⁵⁷) and N (⁵⁵RRR⁵⁷) showed similar motifs, while groups O and P exhibited comparable patterns, (⁵⁵PAA⁵⁷) and (⁵⁵PAR⁵⁷), respectively. Therefore, our results suggest that the structural plasticity of the Tat protein may depend on the ARM region for its optimal functionality. To quantify this variability, Shannon entropy was calculated for residues 49–57 (Fig. 2C, left panel). In group M (*n* = 43,087), residues 49–52 showed near-zero entropy (0.008–0.035), confirming strong conservation of the core RNA-binding segment. Variability was confined mainly to positions 53 (0.253), 54 (0.385), and 57 (0.404), while positions 55 (0.033) and 56 (0.097) remained highly conserved. Despite this diversification, dominant residues preserved electrostatic compatibility (e.g., R53: 77.6%, K53: 18.1%; Q54: 72.6%; R57: 49.5%) (Fig. 2C, right panel). Group N (*n* = 160) was fully conserved across all ARM positions except residue 54 (entropy = 0.167; Q54: 92.5%). Group O (*n* = 546) showed conservation at positions 49–51 (entropy = 0), moderate variability at positions 52–54 (0.075–0.275), and higher entropy at positions 55–57 (0.574–0.671), consistent with its characteristic ⁵⁵PAA⁵⁷ signature. Group P (*n* = 33) exhibited complete conservation (entropy = 0) across the entire ARM. Collectively, these data demonstrate that residues 49–52 are invariant across all four lineages, while diversification at positions 53–57 remains position-specific and structurally bounded, reinforcing that Tat plasticity operates within constraints imposed by preservation of the TAR-binding architecture.

## Structural divergence of Tat–TAR interactions among HIV-1 groups

**Fig. 3:**
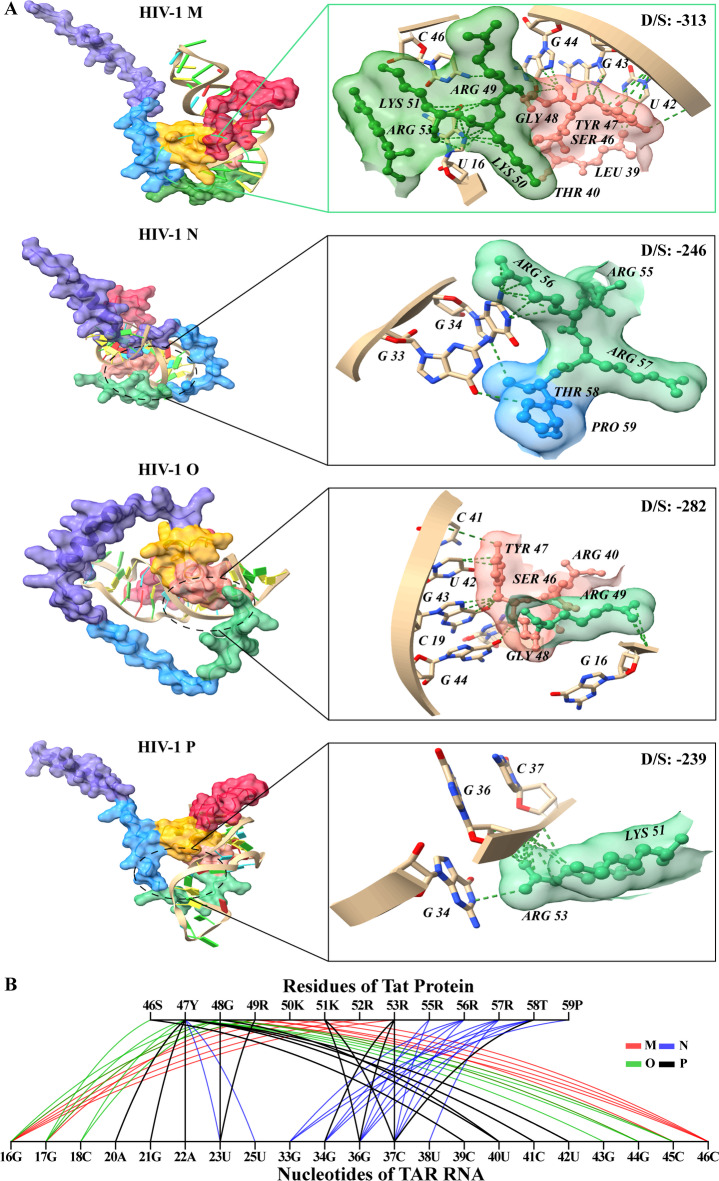
HIV-1 Tat-TAR binding among the groups using molecular docking analysis. (**A**) The binding affinity for groups M, N, O, and P is − 313, − 246, − 282 and − 239 for wild-type Tat-TAR interactions, along with their graphical representation and the interaction network at the ARM region of Tat. (**B**) The interacting residues of HIV-1 Tat with nucleotides of TAR RNA element.

To investigate the functional impact of Tat protein divergence across HIV-1 groups, molecular docking was performed between group-specific Tat consensus sequences and the TAR element using HDOCK. Docking scores (D/S), which are inversely proportional to binding affinity (i.e., more negative values indicate stronger binding), revealed distinct group-specific interactions where group M showed the strongest affinity (–313), followed by groups O (–282), N (–246), and P (–239) (Fig. 3A), while residue–nucleotide interaction patterns are summarized in Fig. 3B. Group M exhibited the most organized and extensive ARM–TAR interface. ARM residues 49R, 50 K, 51 K, 52R, and 53R formed multiple recurrent hydrogen bonds with TAR nucleotides 16G, 17G, 45 C, and 46 C (e.g., 49R–16G, 49R–17G, 49R–45 C, 49R–46 C, 50 K–16G, 50 K–46 C, 51 K–16G, 51 K–46 C, 52R–16G, 53R–16G) (Fig. 3A). As summarized in the residue–nucleotide interaction network (Fig. 3B), these contacts cluster within ARM positions 49–53 and anchor predominantly to TAR nucleotides 16–18 and 44–46, forming a compact and coherent binding interface characterized by dense hydrogen-bond and electrostatic interactions. In contrast, group N displayed a shifted ARM-binding footprint. ARM amino acid residues 55R–57R formed hydrogen bond with TAR nucleotides 33G, 34G, 36G, 37 C, and 38U (Fig. 3B). This down streaming positioning compared to M is likely responsible for reduced binding affinity, consistent with its weaker docking score (Fig. 3A). Group O produced only sparse and localized ARM–TAR contacts (primarily 49R–16G), indicating limited engagement while group P showed a similarly weak and dispersed interaction pattern, with residues 49, 51, and 53 contacting non-contiguous TAR nucleotides (23U, 34G, 36G/C, 37 C), yielding the least stable interface (Fig. 3A, B). The bond density and positional alignment of ARM interactions with specific TAR nucleotides critically shape Tat binding capacity, with group M forming a compact organized interface, whereas groups N, O, and P interact with TAR at fewer or misaligned sites, consistent with their lower binding affinities.

## Co-evolutionary association along the open reading frame of Tat protein of HIV-1 groups

**Fig. 4:**
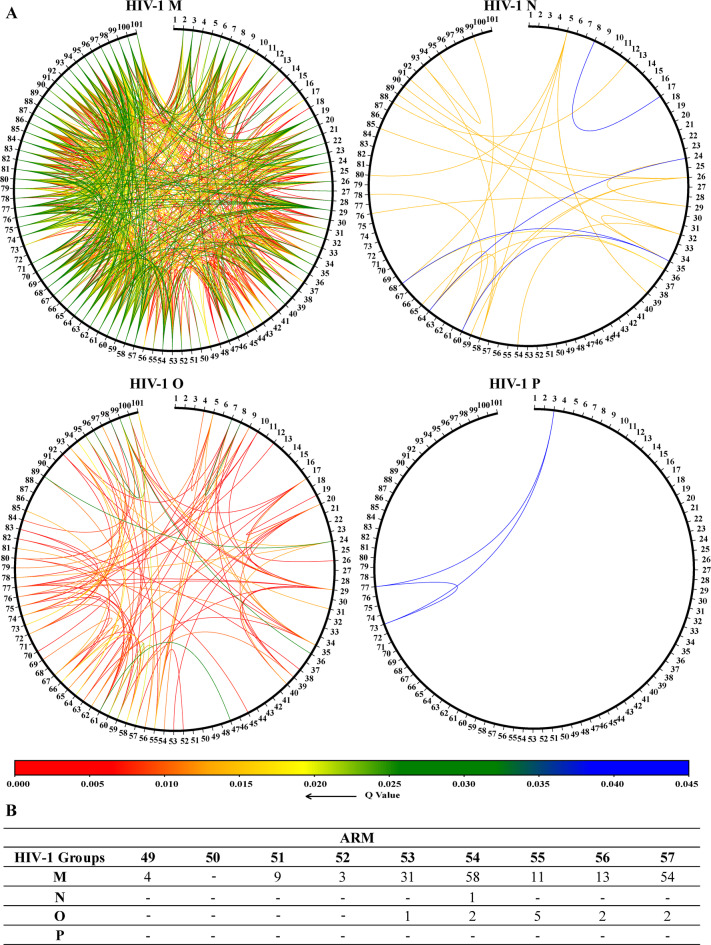
Codon-codon covariation association by Phylogenetic Dependency Network (PDN) analysis. (**A**) HIV-1 groups M, N, O, and P show 1375, 36, 135, and 6 significant codon associations, respectively. Significant associations were defined as q < 0.2 and visualized in a Circos plot. A heatmap color scale is used to represent q-values, with lower q-values (closer to 0; shown in red) indicating stronger statistical significance. (**B**) Number of associations corresponding to each position within the ARM region across different HIV-1 Tat groups.

To explore evolutionary constraints and functional dependencies within the HIV-1 Tat protein, codon covariation analysis was performed using the Phylogenetic Dependency Network (PDN) model across groups M, N, O, and P. Statistically significant covarying associations (q < 0.2) revealed substantial group-specific variation except for group P. Group M exhibited 1,375 significant associations, consistent with its large sequence dataset, global distribution, and complex evolutionary dynamics. Groups N, O, and P showed 36, 135, and 6 significant associations, respectively; the latter were likely limited by smaller sample sizes (Fig. 4A). Within the TAR RNA–binding ARM region, 183 codon–codon associations were identified in group M, forming a dense network of proximal and distal interactions. Key positions 53, 54, and 57 frequently covaried with 101, 46, and 64, respectively (Supplementary Table ST1). Among these associations, the most prominent were 53K_46Y, 54H_64T, and 57S_74P, indicating evolutionary interdependencies that may be required to maintain the protein’s structural stability and functional integrity. Group N showed a single ARM-associated pair, 54R_32F (Fig. 4B), likely due to limited sequence data. In contrast, group O showed 12 ARM-related associations (Fig. 4B), with residues 53, 54, and 55 interacting with positions 20, 93, and 99, respectively. Some interactions were bidirectional (e.g., 52Y_55R and 55R_52Y), suggesting mutual evolutionary dependencies, despite fewer associations than those observed in group M. Due to its small sample size, group P yielded no statistically significant ARM-related associations (Fig. 4B). We observed that the frequencies and patterns of codon co-varying pairs, including the specific amino-acid and positional associations, differed markedly along the Tat ORF, with particularly distinct patterns in the ARM region across HIV-1 groups (Fig. 4B). While group M showed extensive ARM-associated co-evolution (183 associations, including multiple links involving position 49), the same ARM positions in groups N, O, and P displayed few or no associations. This group-specific heterogeneity indicates distinct co-evolutionary selective pressures and highlights the greater flexibility and fitness potential of group M.

## Co-evolutionary compensation and structural adaptation in HIV-1 Tat

**Fig. 5:**
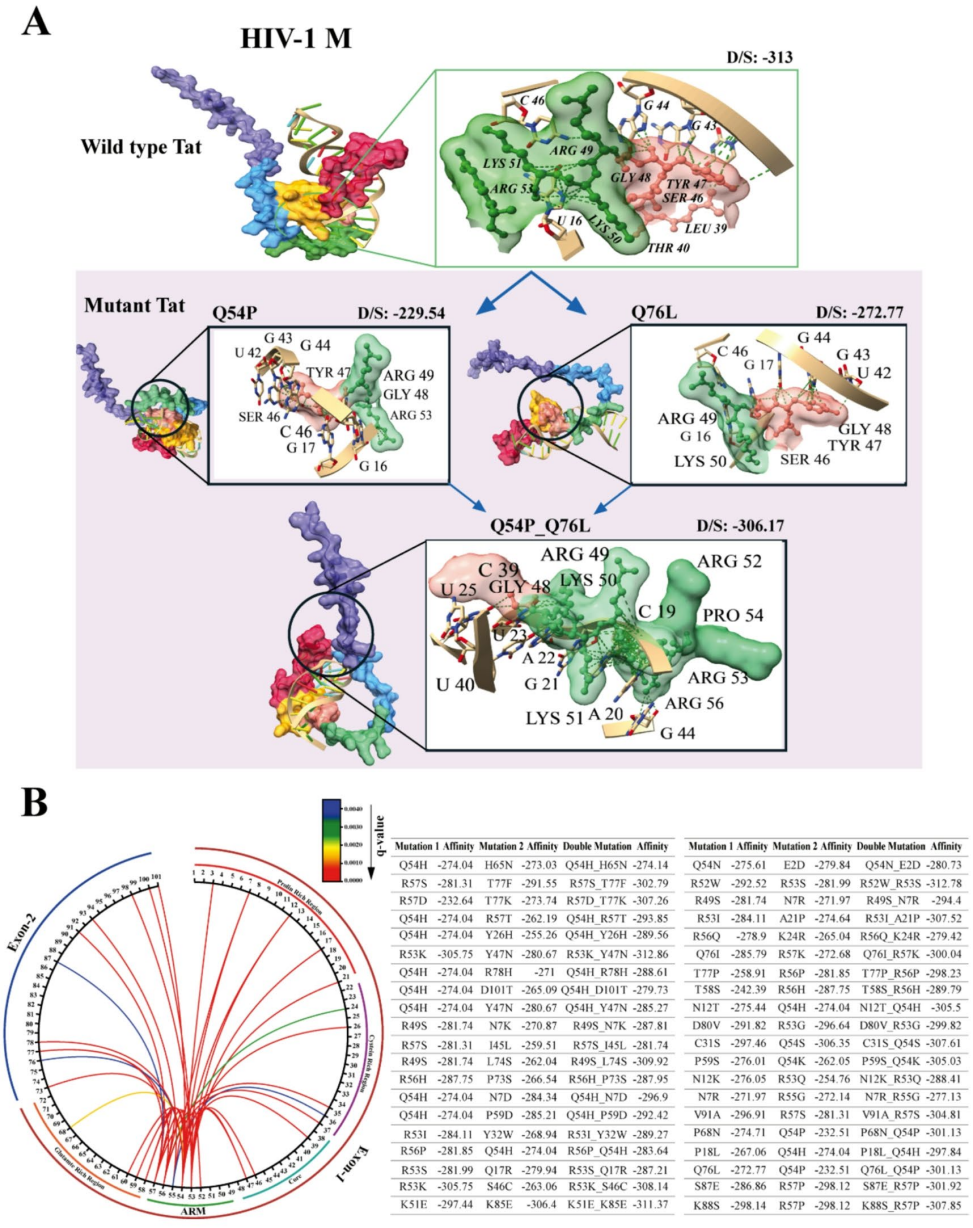
Co-evolutionary Compensation for structural restoration in HIV-1 Tat–TAR complex. (**A**) Molecular docking shows the Tat–TAR interactions for single mutants ∆Q54P and ∆Q76L, and their compensatory double mutant ∆Q54P_Q76L, highlighting partial restoration of binding within the ARM region. (**B**) The circular plot illustrates codon co-covariation between ARM and other Tat regions, identifying 40 compensatory pairs that preserve TAR-binding stability.

To understand the compensatory dynamics of co-evolutionary associations in HIV-1 Tat, we analyzed 1,375 covarying codon pairs from group M and assessed Tat-TAR interactions through molecular docking using HDOCK. Binding interactions were evaluated for both single missense mutants and their associated double mutants relative to the wild-type Tat-TAR complex. Single missense mutations that reduced structural stability and Tat-TAR binding, reflected by higher (less negative) docking scores compared to the wild-type (consensus) structure, were considered deleterious escape mutations^33^. In contrast, double mutants (associated codon pairs) that showed improved docking scores relative to their corresponding single mutants, but not exceeding that of the wild type, were inferred as compensatory mutations, since double mutants with stronger binding than the wild type are biologically improbable^34^. To illustrate compensatory behavior, we selected a co-evolutionary associated pair at amino acid positions 54 and 76. In molecular docking with TAR RNA (*PDB ID*: *1LVJ*), the single mutants ∆Q54P and ∆Q76L exhibited docking scores of −229.54 and −272.77, respectively, whereas the double mutant ∆Q54P-Q76L showed an improved score of −306.17 (Fig. 5A, lower panel). This indicates a partial restoration of binding affinity relative to the wild type (− 313), consistent with compensatory effects without surpassing native Tat-TAR binding strength. (Fig. 5A, upper panel). This pattern highlights the compensatory nature of certain mutational combinations, as revealed by co-evolution in the PDN analysis, which helps maintain the plasticity and functionality of the Tat protein under selective pressure.

From 1,375 covarying codon associations, our analysis identified 355 compensatory mutation pairs in HIV-1 group M Tat within the context of Tat–TAR interactions, 40 of which were located in the ARM region (Fig. 5B). This suggests that HIV-1 Tat may employ co-evolutionary compensation across multiple regions, particularly within crucial functional domains such as the ARM, to preserve structural integrity and transactivation capacity under selective pressure. Overall, evolutionarily linked mutation pairs appear to restore or maintain the structural and functional stability of the Tat–TAR complex, offsetting the deleterious effects of individual substitutions.

### Machine learning–based classification of compensatory mutations in HIV-1 group M Tat

**Fig. 6:**
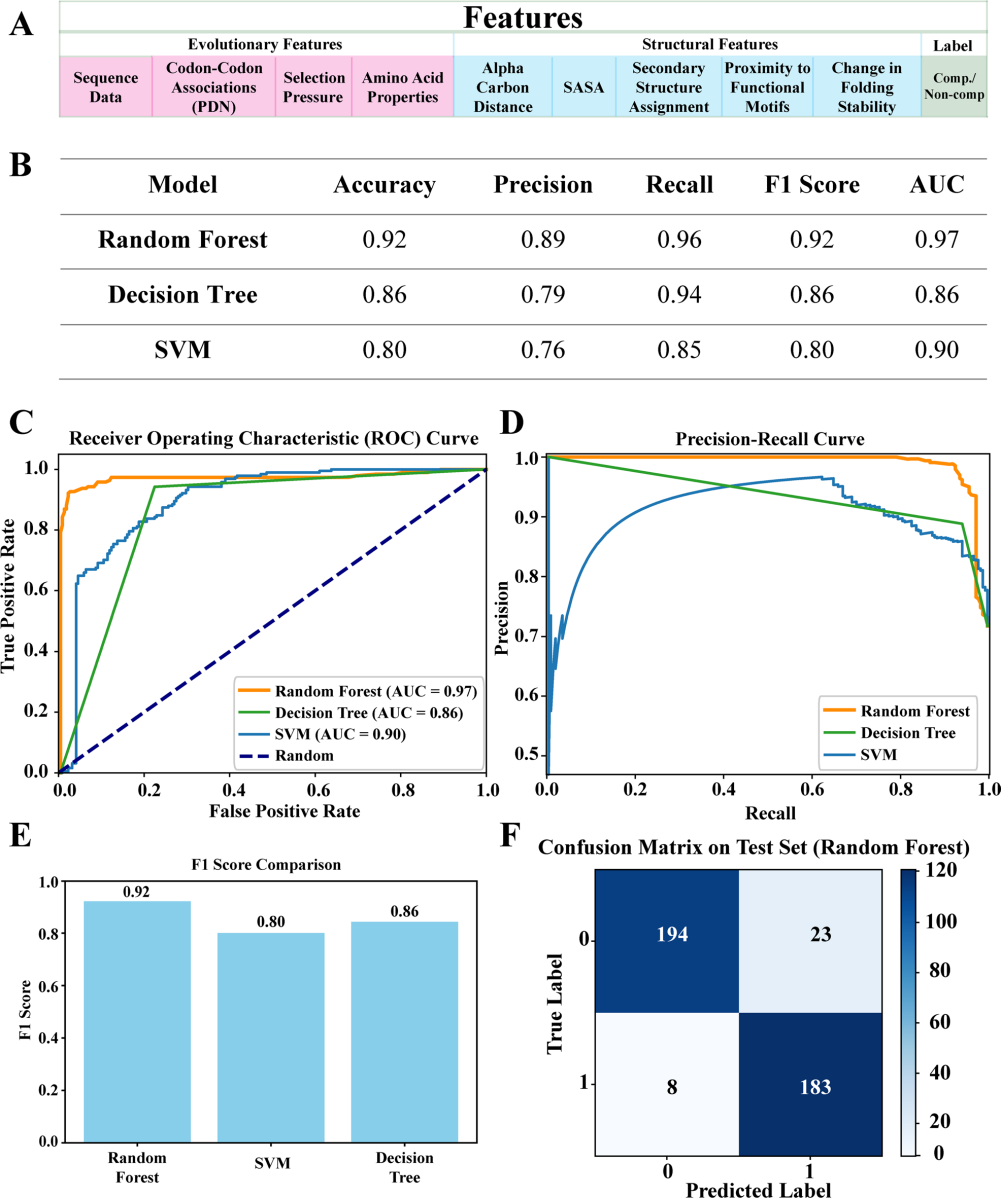
Validation of co-evolutionary compensation through supervised machine learning models. (**A**) Structural and evolutionary features used for model training. (**B**) Performance comparison of three supervised classifiers, Random Forest, SVM, and Decision Tree, based on evolutionary and structural datasets. (**C**) Receiver Operating Characteristic (ROC) curves showing the trade-off between sensitivity and specificity; Random Forest exhibited the steepest curve and highest AUC, indicating superior discriminatory power. (**D**) Precision–recall curves highlighting Random Forest’s consistent performance with high precision and recall, particularly under class imbalance. (**E**) F1-score comparison summarizing the balance between precision and recall across models; Random Forest showed the highest F1 value. (**F**) Confusion matrix visualization of the Random Forest model demonstrating strong true-positive and true-negative predictions, confirming its reliability.

To develop a binary classification system capable of distinguishing compensatory from non-compensatory mutations based on our dataset, we implemented three supervised algorithms: Decision Tree, Support Vector Machine (SVM), and Random Forest. Each model was trained, validated, and tested using structural and evolutionary features derived from 1,375 codon co-variation pairs identified by PDN analysis (Fig. 6A).

Performance evaluation of the supervised models on the independent test set revealed that the Random Forest classifier achieved the highest accuracy (0.92), precision (0.89), and recall (0.96) (Fig. 6B). The Decision Tree model performed moderately (accuracy = 0.86) despite low robustness, while the SVM showed lower accuracy, precision, recall. ROC–AUC analysis confirmed the Random Forest’s superior classification capability (AUC = 0.97), compared to 0.86 for the Decision Tree and 0.90 for the SVM (Fig. 6C). Additionally, Precision–recall and F1-score analyses further supported this, with the Random Forest showing the best balance between precision and recall (Fig. 6D–E).

Further, the confusion matrix of the Random Forest model (Fig. 6F) demonstrated strong predictive reliability, with 194 true negatives, 183 true positives, and relatively few misclassifications (23 false positives, 8 false negatives). Overall, these results validate that ensemble-based classifiers, particularly Random Forest, can accurately learn compensatory mutation patterns from large-scale association data. Such models may be extended for future predictive applications in identifying functionally relevant compensatory mutations and prioritizing therapeutic targets in HIV-1 evolution studies.

## Discussion

The exceptional evolutionary plasticity of HIV-1, driven by co-evolutionary adaptation and complex mutational dynamics, continues to present a formidable barrier to effective vaccine development. This mutational plasticity, while contributing to the remarkable sequence variability observed across global HIV-1 lineages, also influences their distinct geographic distributions and pathogenic outcomes. Among the major HIV-1 groups (M, N, O, and P), group M, with its numerous subtypes including circulating recombinant forms (CRFs), and unique recombinant forms (URFs), remains responsible for the ongoing global HIV/AIDS pandemic^35^. Understanding these evolutionary dynamics, which balance viral adaptation against fitness costs imposed by host selection, is therefore critical for therapeutic design. Mutations arising under selective pressure often compromise viral fitness, yet are frequently followed by compensatory substitutions that restore functionality, ensuring viral persistence despite immune and therapeutic constraints^36–38^.

We have analyzed a global dataset of over 43,000 HIV-1 *tat* gene sequences collected over 25 years (1997–2021), including groups M (*n* = 43,087), N (*n* = 160), O (*n* = 546), and P (*n* = 33). Because group M is responsible for the global HIV-1 pandemic, its significantly larger representation in public databases reflects biological and epidemiological reality rather than analytical bias. Therefore, the difference in dataset sizes accurately represents the true distribution of circulating viral diversity among HIV-1 group M, N, O, and P. To address potential sampling overrepresentation, all evolutionary analyses were performed separately within each group to avoid numerical bias from pooling. In addition, group M was randomly downsized to 739 sequences, matching the total number of non-M sequences, and the analyses were repeated. The overall pattern of positive selection (dn/ds > 1.0), entropy distribution, and phylogenetic clustering remained unchanged after down sampling (Supplementary Fig. SF1). These findings confirm that the adaptive signatures observed in Tat are not driven by sampling imbalance but instead reflect genuine lineage-specific evolutionary pressures acting naturally at the population level.

Some of the previous studies have examined selective pressure and amino acid variability in various HIV-1 proteins under host-mediated evolution^32,39,40^. Our research group has also reported the evolutionary dynamics of the HIV-1 Vif protein, highlighting how selection pressure shapes viral mutational dynamics from the circulating strains of HIV-1 globally^35^. However, analyses of the *tat* gene have so far been limited to specific HIV-1 subtypes (i.e., type B and C) and timeframes^31,32^, lacking a comprehensive understanding of group-specific evolutionary patterns in a global context. For example, a prior regional study analyzed 120 clinical samples from North India and reported 11 natural mutations within exon-1 of Tat^31^. Rather than cataloging mutations in a localized cohort, we quantified mutation frequencies across the complete Tat ORF at a global population scale. Additionally, our study integrates co-evolutionary associations and structural compensation mechanisms to provide a broader picture of Tat’s mutational plasticity across all four major HIV-1 groups: M, N, O, and P.

Tat is one of the most critical regulatory genes of HIV-1 and plays a central role in viral replication, pathogenesis, and disease progression^41^. Tat amplifies viral gene expression, leads to high levels of structural proteins (Gag, Pol, Env), and efficient production of new virions^42^. Inhibitors targeting the Tat protein, such as dCA, have been shown to disrupt the interaction between Tat and TAR, effectively blocking viral replication^43^. The role of the Tat protein in HIV-1 pathogenesis is well documented. Functional assays conducted by Ronsard et al. and Raja et al. have examined Tat in the context of subtype-specific phenotypes, focusing on factors such as RNA interference silencing suppressor (RSS) activity^44^ and Tat’s modulation of host factors, including the stabilization of Mdm2^45^. While previous studies focused on subtype-specific functional mechanisms, particularly how Tat enhances viral replication through interactions with host factors, our study takes a structural and evolutionary approach. We examine population-level diversification and compensatory codon networks within the Tat–TAR interface, independent of subtype differences or geographic boundaries.

Globally circulating HIV-1 strains continue to evolve under intense host immune pressure, as reflected by elevated nonsynonymous substitution rates (dn) relative to synonymous rates (ds), indicating adaptive rather than neutral evolution. Our comparative analysis shows that group M exhibits the most consistent dn/ds ratio (2.20) alongside moderate entropy (0.21), setting it apart from groups N, O, and P (dn/ds ratio: 2.04, 2.45, and 1.08; entropy: 0.21, 0.35, and 0.01, respectively) (Fig. 1A, C). Statistical comparisons further demonstrated that groups M and N (*r* = 0.39, *p* < 0.05) share closer evolutionary patterns than other group pairs (*r* = 0.66 and *r* = 013; *p* > 0.05) (Fig. 1B, D), which likely reflects their more similar host origin simian immunodeficiency virus (SIVcpz) found in the central chimpanzee subspecies (*Pan troglodytes troglodytes*)^46–48^. While the initial divergence of HIV-1 groups arose from distinct cross-species transmissions, M and N from chimpanzees, O and P from gorillas, M and N appear to have gradually converged under human-driven selective forces. In contrast, groups O and P have continued to evolve along distinct trajectories shaped by unique ecological and host-specific contexts^46–48^. Such evolutionary stability in group M suggests that Tat evolves under persistent selection pressure but is buffered by compensatory mechanisms that restore or preserve fitness, thereby maintaining transcriptional potency and supporting its global predominance.

Structural comparisons among the groups revealed notable variations in the Tat protein, particularly in the arginine-rich motif (ARM), which is crucial for TAR RNA binding (Fig. 2A). Despite overall conservation of the canonical ARM sequence (^49^RKKRRQRRR^57^) group-specific amino acid signatures were evident, especially at positions 55 to 57, where groups M (^55^RRR/G/S^57^) and N (^55^RRR^57^) showed similar motifs, while groups O and P exhibited comparable patterns, (^55^PAA^57^) and (^55^PAR^57^), respectively. (Fig. 2A-B). Although the precise backbone conformations of the ARM differ among the variants (Fig. 2A), the clustering and surface exposure of positively charged residues are maintained, suggesting preservation of the region’s electrostatic properties. The AlphaFold2-predicted full-length models showed moderate global confidence (average pLDDT: M 70.02; N 70.04; O 70.38; P 71.48), whereas the ARM region exhibited comparatively lower local confidence scores, consistent with intrinsic disorder, sequence variability, and the absence of an experimentally resolved full-length Tat structure in the PDB. Only truncated Tat fragments (e.g., *PDB IDs: 1JFW, 1K5K, 1TIV*) were available as partial template hits during modeling, which may further limit structural certainty in flexible segments. Thus, our predicted models should be interpreted as representative conformations derived from computational prediction rather than definitive structures, particularly for the flexible ARM region (Fig. 2B). The subtle differences among the groups may modulate Tat–TAR binding affinity and transcriptional activation efficiency, and our observation is consistent with the other mutational study in Tat basic domain in vivo^18^.

Consistent with this, our molecular docking confirmed that the group M Tat exhibited the strongest TAR-binding affinity (D/S: − 313), underscoring its superior functional capacity compared to the other groups (Fig. 3A). These findings suggest that conservation of ARM structural integrity is central to Tat’s transcriptional potency and likely may underlie group M’s evolutionary advantage, whereas divergence in this region among HIV-1 groups N, O, and P may contribute to their lower replication efficiency, and could also help explain their highly restricted geographic distribution: groups N has been documented almost exclusively in Cameroon^49^, by contrast O, is found at low but stable prevalence mainly in West-Central Africa, especially Cameroon, Gabon, Chad, Nigeria, Togo, and Senegal^50^, and P is extremely rare, having so far been reported only in a few individuals in Cameroon^51^.

Tat–TAR interactions are known to be disrupted by mutations within the ARM, leading to reduced TAR binding, impaired nuclear import, and diminished transcriptional activation^21–27^. Importantly, mutations rarely occur independently under selection pressure; instead, they arise in a coevolutionary manner, where a substitution at one site influences another^52^. To explore such dependencies, we applied the Phylogenetic Dependency Network (PDN) model^53^ to infer codon covariation across Tat. Co-evolution in other HIV proteins, such as gag, env, vpu, shows similar patterns; for example, Gag contains structurally proximal, functionally compensatory amino-acid pairs^39,54^, clustering analyses reveal recurrent co-occurring mutations indicative of epistasis^55^, and integrated prediction methods identify coevolving intra-protein and inter-protein sites (e.g., Gag–protease under drug pressure) located in interacting functional regions^56^, supporting the relevance of the codon-pair dependencies we report. Our analysis identified 1,375 covarying associations in group M, 36 in N, 135 in O, and only 6 in P (Fig. 4A). Within the ARM, 183 associations were detected in group M, indicating a particularly dense co-evolutionary network. This extensive network implies that although group M accumulates mutations rapidly, structural integrity is preserved through compensatory co-adaptations, sustaining Tat’s function and pathogenic potential^33^.

Our previous study on HIV-1 Vif also revealed an oscillatory escape mutation-reversion cycle, one of the mechanisms by which the virus might regain fitness^35^. Interestingly, analogous compensatory behavior has been observed in Tat, where co-varying paired substitutions (e.g., Q35L/I39Q) can restore transcriptional activity to near wild-type levels^33^. Co-immunoprecipitation further confirmed that these residues affect distinct steps in Tat function, residue 35 is critical for P-TEFb binding, while residue 39 influences RNAPII phosphorylation, demonstrating true compensatory interaction between the two sites^8,57,58^. Similarly, although prior functional study^59^, characterized individual mutations such as S46F and S61R and demonstrated enhanced TAR interaction, our approach extends beyond single-site functional characterization by identifying statistically supported co-evolving mutation networks. For example, S46F was associated with Y47F and G83D, while S61R co-varied with N67A, D98H, and H65N (Supplementary Table-ST1), revealing adaptive mutational coupling and epistatic compensation within Tat. In this study, by integrating PDN-inferred associations with molecular docking validation, we identified 355 mutation pairs with compensatory potential (Supplementary Table-ST1), including 40 within the ARM (Fig. 6B). Importantly, while earlier reports described mutations such as R52W and speculated on their functional consequences^31^, our integrative structural and co-evolutionary analysis demonstrates that R52W impairs TAR interaction and is statistically coupled with a compensatory substitution at position 53 (R53S). This indicates that when tryptophan emerges at position 52, serine at position 53 becomes overrepresented, suggesting coordinated adaptive compensation at the population level. Such compensatory pairing was not evaluated in previous regional analyses and underscores the importance of large-scale evolutionary coupling frameworks. Nevertheless, these findings are derived from computational and population-level analyses, and direct experimental validation in the wet laboratory will be essential to confirm the functional interplay and compensatory mechanism between R52W and R53S. This highlights Tat’s capacity to retain functional stability through structural co-adaptation, a mechanism that may be pivotal to the persistence of group M variants worldwide.

Compared with prior ML/DL studies in HIV-1 that focused on predicting drug resistance^60^, infection status from clinical features^61^, or subtype classification using sequence embeddings^62^, our approach is fundamentally different in both objective and methodology as no previous work has performed mutational classification (compensatory vs. non-compensatory) using an integrated framework of evolutionary and structural features^53^. By directly linking evolutionary coupling, structural context, and mutation outcome, our method provides a mechanistically grounded prediction strategy that goes beyond sequence-only or phenotype-only ML approaches, highlighting the novelty and added biological interpretability of our work. Among the tested algorithms, the Random Forest model achieved the highest performance (accuracy = 0.92, F1 = 0.92, AUC = 0.97; Fig. 6B), outperforming Decision Tree and SVM classifiers. This suggests that Tat’s compensatory dynamics can be computationally captured through integrated feature sets, including selection pressure, co-evolutionary patterns, and structural metrics.

Although we did not explicitly separate escape and reversion events, the model effectively distinguished compensatory mutations, reinforcing the notion that Tat maintains its transactivation capacity through a balance of deleterious and restorative mutations. In addition, our integrative framework combines phylogenetic dependency network (PDN) inference, structural modeling, molecular docking, and machine learning classification, the majority of predicted compensatory mutation pairs were not experimentally validated. While docking simulations and evolutionary coupling analyses provide supportive structural and statistical evidence, they cannot fully substitute for in vitro or in vivo functional assays measuring Tat transactivation, TAR binding affinity, or P-TEFb recruitment. Accordingly, the compensatory potential of many identified mutation pairs remains computationally inferred. Even though our findings provide mechanistic insights into Tat’s evolutionary adaptability, the translational implications remain speculative. Functional replication assays, host-factor interaction studies, and therapeutic targeting experiments will be required to determine whether identified compensatory networks can be exploited for antiviral intervention.

Overall, our findings reveal that Tat’s evolutionary adaptability is maintained through structural and co-evolutionary compensation, particularly within the ARM. These compensatory associations buffer the impact of deleterious mutations, preserving Tat–TAR binding and transcriptional activity under host selective pressure. Understanding these dynamics advances our view of Tat’s molecular evolution while also guiding future antiviral strategies targeting Tat’s conserved motifs and compensatory networks. Although Tat’s evolutionary path does not fully mirror broader HIV evolution, the patterns align with known HIV-1 polymorphisms, offering insights that could inform future therapies targeting Tat’s antagonistic function, resilient to mutational escape.

## Conclusion

HIV-1 Tat demonstrates group-specific adaptation, with group M exhibiting the highest levels of mutational plasticity and TAR RNA binding affinity. This underscores its enhanced adaptability and structural plasticity. In group M, codon co-evolution reveals extensive networks of compensatory mutations that maintain Tat’s structural and functional stability under selective pressure. By employing supervised machine learning (ML), we can predict these compensatory interactions among mutations, thereby establishing a framework to identify the evolutionarily constrained and functionally critical sites within Tat. The integration of AI-driven computational approaches provides a powerful method for rapidly detecting mutational dependencies, which accelerates the discovery of new therapeutic targets and informs the rational design of interventions aimed at critical positions in Tat. This methodology can also be applied to other HIV-1 proteins that can address the virus’s extensive genetic diversity. Therefore, our study represents a foundational step toward decoding Tat’s global mutational landscape and leveraging evolutionary principles for the design of antiviral strategies.

## Materials and methods

### Sequence retrieval, processing and dataset curation

HIV-1 *tat* gene sequences (*n* = 45,183) from 1997 to 2021 were retrieved from the Los Alamos National Laboratory (LANL) HIV Sequence Database (http://www.hiv.lanl.gov/). Sequences were first downloaded separately for groups M, N, O, and P using web alignments approach (https://www.hiv.lanl.gov/content/sequence/NEWALIGN/help.html#web), which is comprehensive, allowing only one sequence per patient. Very similar sequences are deleted based on predetermined cut-offs established by analyzing distance distributions, which vary by gene along with the removal of any problematic sequences, and subsequently curated to yield final datasets of M (*n* = 43,087), N (*n* = 160), O (*n* = 546), and P (*n* = 33). All processing steps were performed using the Biopython v1.81 package within custom Python scripts^35,63^. After that, sequences were further processed to remove gaps and aligned with the reference consensus sequence using the Muscle algorithm in MEGA11^64^, and subsequently translated into protein sequences. Missing consensus sequences were generated using Geneious Prime v2023.1.1^65,66^.

### Analysis of HIV-1 Tat variability under host-mediated selection pressure

The immune-mediated selection pressure on HIV-1 *tat* genes was evaluated using SNAP v2.1.1 tools from the LANL database^67^. At each of the 101 codons in Tat, SNAP examined the ratios of synonymous (ds) and non-synonymous (dn) substitutions. A value equals to 1 indicates neutral selection and greater than 1 denotes positive or natural selection. On the other hand, a dn/ds < 1 indicates negative or purifying selection^67,68^.

To assess the selection pressure on protein level, Entropy one tools in LANL database (https://www.hiv.lanl.gov/content/sequence/ENTROPY/entropy_one.html) was used to assess the amino acid variability of HIV-1 Tat protein. Shannon entropy score for each 101 amino acid position of Tat protein was computed^10^ and a bash scripting with web scrapping were used to automate the process. A maximum-likelihood phylogenetic tree was generated for HIV-1 Tat sequences of groups M, N, O, and P using FastTree^69^, and the resulting tree was visualized and annotated with group-specific coloring in iTOL^70^.

### Sequence conservation of HIV-1 Tat protein

Sequence logos represent the variability from the multiple sequence alignments, with stack height indicating conservation and height of each letter, showing the relative frequency of each amino acid. To assess sequence conservation of Tat across the entire open reading frame (ORF), we used Matplotlib^71^ and Web Logo package^72^, through a custom Python script to generate sequence logos from our HIV-1 Tat sequences dataset comprising 25 years of sequences (1997–2021; *n* = 45,183).

### Analysis of co-evolutionary association within HIV-1 Tat protein

Codon–codon covariation identifies statistically significant relationships between mutations at different sites, reflecting functional constraints, and codon co-evolution, where a mutation at one position influences changes at another to preserve structure or function under evolutionary pressure. In HIV-1 Tat, such covariation reveals mutation pairs, often located at binding interfaces, that help maintain structural integrity, sustain TAR RNA interactions, and support overall viral fitness. The Phylogenetic Dependency Network (PDN) model from Carlson et al. 2008^53^ was modified and adjusted to analyze the significant codon association through Fisher’s Exact test based on co-occarance patterns. This method systematically compares every possible amino acid position between two codons using the features of TT (Presence-Presence), TF (Presence-Absence), FT (Absence-Presence), and FF (Absence- Absence) using a 2 × 2 table, followed by multiple-testing correction with Tibshirani’s False Discovery Rate (FDR) approach within our study context. A threshold of q value, q < 0.2, was applied to select significant associations and was visualized in a circus plot using a customized Python script based on a Circos model package^73^.

### Structural prediction of HIV-1 Tat protein

Structure of HIV-1 Tat protein, including those from M, O, N, P groups, was predicted from representative consensus amino acid sequences, using AlphaFold2 via ColabFold^74–76^. The structure of the mutant Tat protein, that deemed significant in coevolutionary analysis, was also predicted. All sequences corresponded to the full-length Tat protein (101 amino acids) with the template mode set to pdb100, enabling the use of known structural homologs. For each input sequence, the top-ranked model (rank_001) based on the internal AlphaFold confidence metric (pLDDT) was selected for downstream analysis. No structural refinement was performed to avoid force-field-specific artifacts and preserve sequence-driven predictions for unbiased comparison across Tat variants^74–80^. The resulting models were visualized and annotated using UCSF ChimeraX^81^.

### Molecular docking of HIV-1 Tat and TAR RNA element

Molecular docking is a computational method used to predict how two molecules, typically a protein and a ligand or two interacting proteins or a protein and a nucleic acid, bind to each other^82^. It estimates the preferred binding orientation, interaction interface, and binding affinity based on shape complementarity, energetics, and structural constraints. We performed molecular docking to assess HIV-1 Tat binding to TAR RNA, using wild-type variants for intergroup comparison and mutants to examine putative compensatory changes^83^. The structure of the 31-nucleotide TAR RNA was obtained from the Protein Data Bank (*PDB ID: 1LVJ*), which was chosen because its bulge region, critical for Tat recognition, is well preserved^84^. Each Tat variant was docked with TAR RNA using the HDOCK server with default settings, grid spacing controlling translation and Euler angles guiding rotation. Receptor (Tat) and ligand (TAR RNA) coordinates were centered for accurate placement. HDOCK produced multiple binding modes, scored by predicted energy and RMSD, allowing identification of the most favorable Tat-TAR interactions^85^. Docking scores were analyzed to assess the effects of specific mutations on the Tat-TAR interaction, providing insights into how codon coevolution may influence structural and functional plasticity.

### Classification of compensatory mutations with machine learning

Leveraging data on selection pressure, entropy, PDN-based co-evolutionary associations, structural features, and binding affinities between wild-type and mutant HIV-1 Tat–TAR RNA complexes, we developed a machine-learning framework to classify compensatory mutation. In this study, a compensatory mutation is defined as a statistically significant associated mutation that may arise following a host-mediated escape mutation in a codon, partially restoring wild-type–like functionality (i.e., Tat-TAR binding affinity) without exceeding the wild-type level, thereby reflecting functional compensation in a co-evolutionary context. Our dataset comprised 914 amino acid mutation pairs, labeled as compensatory^1^ or non-compensatory (0) based on the binding affinities of Tat-TAR complexes represented by docking scores. For each associated mutation pair, feature extraction encompassed both evolutionary and structural parameters. Evolutionary features included mutated amino acid sequences^86,87^, codon co-occurrence patterns (TT, TF, FT, FF)^53^, site-specific selection pressure (dn/ds) and entropy scores. Structural features included Cα–Cα distances, physical proximity, solvent accessible surface area (SASA)^88^, secondary structure assignments, functional region annotations^89^, and predicted ΔΔG stability^90^. Single missense mutants with reduced binding affinity (reflected by higher, less negative docking scores) indicated disruption of native Tat–TAR interaction, while double mutants were evaluated for restoration of binding. To mitigate class imbalance between compensatory and non-compensatory mutations, we applied random oversampling, duplicating minority-class instances until classes were balanced^43,91^. This ensured unbiased model training and improved classification performance. Supervised classifiers, including Random Forest (RF), Support Vector Machine (SVM), and Decision Tree (DT) were implemented in Python using scikit-learn. Feature vectors spanned engineered attributes, with labels indicating compensatory status. The RF model employed 100 trees (n_estimators = 100) with a fixed random seed (random_state = 42) for reproducibility. Model performance was evaluated across independent training (70%), validation (10%), and test (20%) datasets. Accuracy metrics and confusion matrices were computed and visualized to assess predictive performance and generalization. This integrative approach enabled the identification of functionally relevant compensatory mutations in HIV-1 Tat, linking co-evolutionary patterns to structural and functional plasticity, and providing mechanistic insights into viral transcription regulation. (Ref: Supplementary Methods for detailed feature engineering, encoding strategies, and model implementation).

### Statistical analysis and machine learning algorithms

Statistical analyses were performed using GraphPad Prism 9.0.0 (GraphPad Software, Boston, MA, USA, www.graphpad.com) and Python. The Mann–Whitney U test was used to compare differences between two independent groups when the data were not normally distributed, and the Spearman rank-order correlation was applied to assess monotonic relationships between variables. Fisher’s exact test and the Storey–Tibshirani procedure were implemented using custom Python scripts. An asterisk (*) indicates a significance level of *p* < 0.05. For predictive modeling, several machine learning algorithms were employed: Random Forest, an ensemble method that builds multiple decision trees and aggregates their predictions for robust classification; Support Vector Machine (SVM), which identifies an optimal hyperplane to separate classes in high-dimensional space; and Decision Tree, a model that recursively partitions data based on feature values to make predictions. Models were trained and evaluated using Python’s scikit-learn library, with performance assessed through cross-validation and standard metrics.

## Supplementary Information

Below is the link to the electronic supplementary material.

Supplementary Material 1

## Data Availability

The machine-learning Python scripts, including those used for compensatory mutation classification, are available in a GitHub repository (https://github.com/Ridwanul-Karim/Compensatory-Mutation-Classifier-of-HIV-1-Tat-Protein). The sequence datasets, scripts supporting the findings of this study are available from the corresponding author upon reasonable request.

## Abbreviations

HIV-1: Human immunodeficiency virus type-1
Tat: Trans-activator of transcription
TAR RNA: Trans-activation response RNA element
LANL: Los alamos national laboratory
dn/ds: Non-synonymous vs. synonymous substitution rate ratio
PDN: Phylogenetic dependency network
D/S: Docking score
ML: Machine learning
RF: Random forest
SVM: Support vector machine
DT: Decision tree

## Declaration

Declaration of generative AI and AI-assisted technologiesThe authors used GPT-5 to enhance clarity and readability. All content was carefully reviewed and edited by the authors, whoassume full responsibility for the final manuscript.
