# Supplementary material for: Predicting the evolutionary and structural compensation in Tat ARM region across HIV-1 groups using machine learning approach
Source: Sci Rep. 2026 Apr 24;16:18919. doi: 10.1038/s41598-026-49324-2 (PMC13276188; doi:10.1038/s41598-026-49324-2)
Supplement: Supplementary file 1 — Supplementary Material 1 [file 41598_2026_49324_MOESM1_ESM.docx]

**Predicting the Evolutionary and Structural Compensation in Tat ARM Region Across HIV-1 Groups using Machine Learning Approach**

*Ridwanul Karim^#^, Md Sakil Arman^#^, K.M. Kamrul Hasan, Zafrul Hasan^*^*

Department of Biochemistry and Molecular Biology, Shahjalal University of Science and Technology, Sylhet- 3114, Bangladesh

^*^Corresponding Author

^#^Authors Contributed Equally

Zafrul Hasan, PhD

Associate Professor

Phone: +88-01706247124

Email: [zafrul-bmb@sust.edu](mailto:zafrul-bmb@sust.edu)

**Supplementary**

**
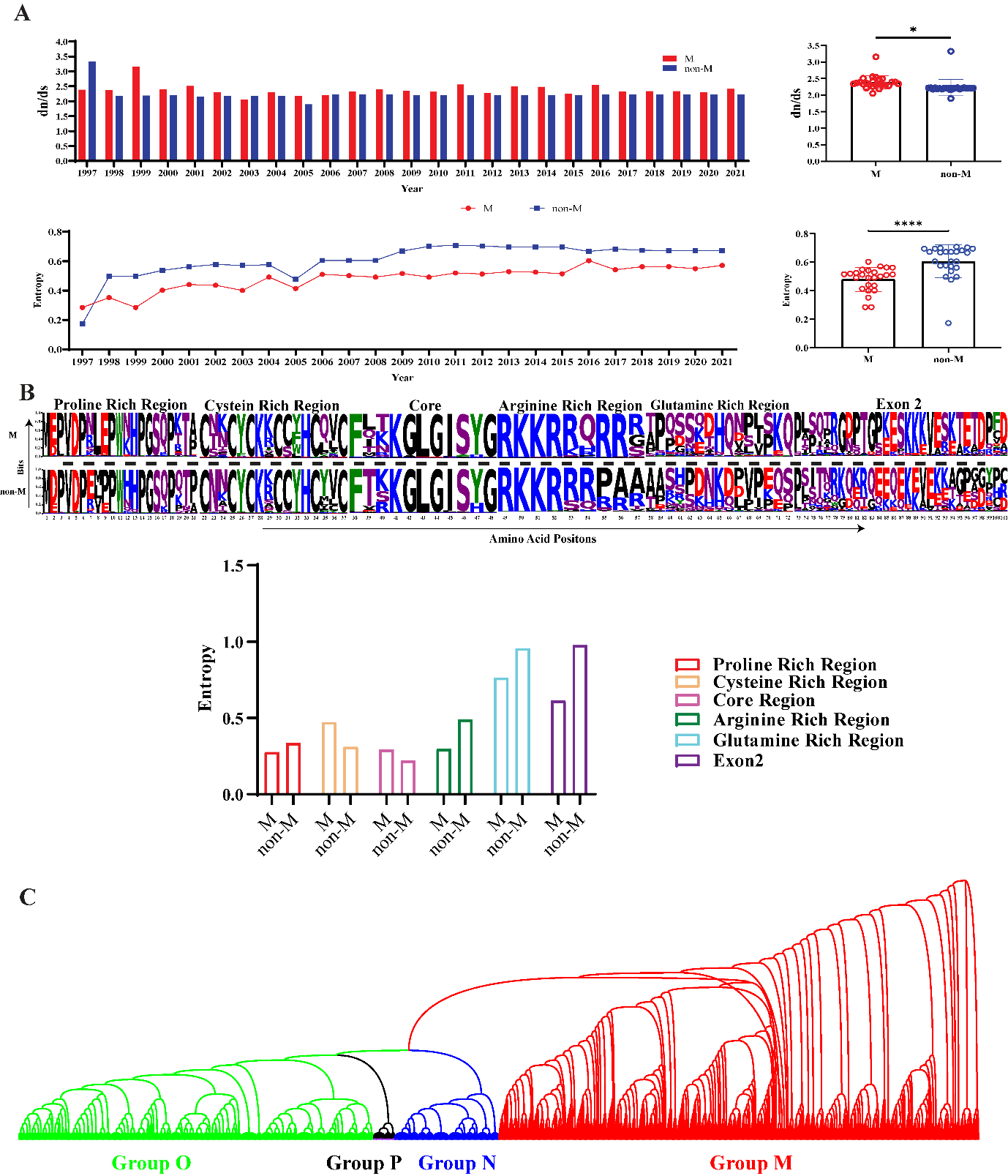
**

**Supplementary Fig. SF1:** **Comparative analysis of selection pressure among HIV-1 groups M, N, O, and P with down-sampled dataset.** (A) Selection pressure was calculated using dn/ds and entropy at the gene and protein level between M (n=739) and non-M (n=739, composed of N [160], O [546] and P [33]) group of HIV-1, where *p<0.05 and ****p<0.0001. (B) Sequence logo representation of amino acid variability along the TAT open reading frame in upper panel, where lower panel showing the entropy as a quantitative measurement of variability between the groups for each domain. (C) Phylogenetic analysis among the HIV-1 group M (n=739), N (n=160), O (n=546) and (P=33).


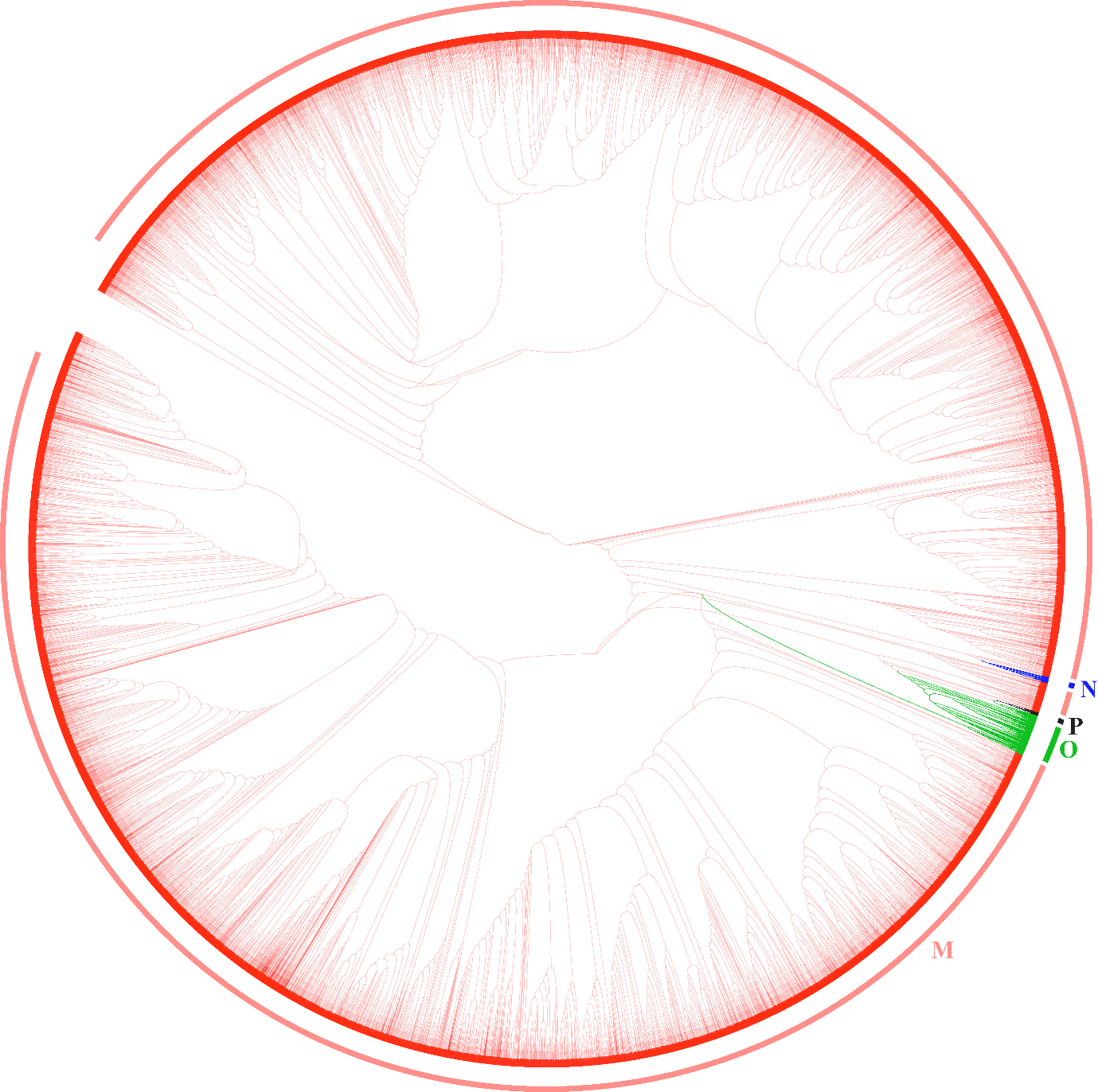


**Supplementary Fig. SF2: Phylogenetic tree of M, N, O, and P of HIV-1 groups.** HIV-1 sequences were retrieved from LANL (n=43,826) and among them M = 43,087, N = 160, O = 546, and P = 33.

**Supplementary Table ST1: Compensatory mutations among associations.**

| Mutation 1 | Affinity | Mutation 2 | Affinity | Double Mutation | Affinity |
| --- | --- | --- | --- | --- | --- |
| L39Q | -290.56 | D101H | -268.05 | L39Q_D101H | -290.83 |
| L39I | -275.65 | K29H | -271.18 | L39I_K29H | -287.11 |
| S93R | -282.4 | D80I | -273.04 | S93R_D80I | -300.97 |
| Q54H | -274.04 | H65N | -273.03 | Q54H_H65N | -274.14 |
| K19T | -295.79 | S93T | -299.39 | K19T_S93T | -307.34 |
| P84S | -280.88 | D64I | -279.09 | P84S_D64I | -291.87 |
| Q63E | -264.72 | S62D | -265.66 | Q63E_S62D | -287.12 |
| L74A | -290.96 | D80I | -273.04 | L74A_D80I | -298.18 |
| C31S | -297.46 | Q60E | -281.96 | C31S_Q60E | -303.94 |
| P84G | -273.64 | G83T | -283.31 | P84G_G83T | -286.9 |
| G83T | -283.31 | K85P | -289.38 | G83T_K85P | -300.63 |
| P3L | -302.01 | K24P | -269.52 | P3L_K24P | -306.04 |
| Q76I | -285.79 | D64I | -279.09 | Q76I_D64I | -299.7 |
| Q76I | -285.79 | D101N | -300.93 | Q76I_D101N | -312.15 |
| E92A | -280.46 | N12Q | -288.72 | E92A_N12Q | -292.14 |
| R57S | -281.31 | T77F | -291.55 | R57S_T77F | -302.79 |
| L74I | -300.04 | S75T | -302.51 | L74I_S75T | -312.26 |
| T20N | -278.89 | P3M | -286.02 | T20N_P3M | -289.31 |
| S87E | -286.86 | D101F | -278.1 | S87E_D101F | -301.57 |
| Q35K | -258.56 | C31N | -266.05 | Q35K_C31N | -270.32 |
| C31R | -264.52 | Y32R | -264.99 | C31R_Y32R | -273.02 |
| T40N | -274.25 | K24Q | -266.1 | T40N_K24Q | -308.19 |
| G83D | -280.55 | K29Q | -271.81 | G83D_K29Q | -305.07 |
| P84S | -280.88 | P68A | -291.36 | P84S_P68A | -294.69 |
| C30Y | -256.53 | P68F | -248.45 | C30Y_P68F | -273.96 |
| T77L | -265.21 | K19A | -278.15 | T77L_K19A | -284.37 |
| Q35K | -258.56 | H33Y | -265.73 | Q35K_H33Y | -271.95 |
| Q35K | -258.56 | Y32C | -259.48 | Q35K_Y32C | -276.13 |
| N7H | -283.52 | G48C | -270.16 | N7H_G48C | -291.02 |
| Q35F | -279.96 | C25S | -273.76 | Q35F_C25S | -293.6 |
| Q60P | -264.92 | P68Y | -292.56 | Q60P_P68Y | -302.55 |
| E96H | -264.48 | T95P | -286.38 | E96H_T95P | -299.47 |
| C27S | -269.5 | A21S | -277.46 | C27S_A21S | -279.82 |
| E86A | -293.85 | K85D | -279.44 | E86A_K85D | -303.2 |
| G83D | -280.55 | S93T | -299.39 | G83D_S93T | -307.3 |
| G79K | -288.26 | E92K | -265.06 | G79K_E92K | -288.31 |
| R57D | -232.64 | T77K | -273.74 | R57D_T77K | -307.26 |
| Q76I | -285.79 | Q60E | -281.96 | Q76I_Q60E | -288.9 |
| Y32G | -263.65 | P18H | -269.11 | Y32G_P18H | -274.77 |
| Y32W | -268.94 | Y47N | -280.67 | Y32W_Y47N | -287.71 |
| Q76I | -285.79 | N12Q | -288.72 | Q76I_N12Q | -304.7 |
| P81H | -276.78 | T97E | -276.06 | P81H_T97E | -296.81 |
| F100D | -291.84 | T97P | -291.5 | F100D_T97P | -296.37 |
| P68L | -289.73 | T77F | -291.55 | P68L_T77F | -298.47 |
| E92T | -268.91 | L74R | -248.76 | E92T_L74R | -277.44 |
| E96H | -264.48 | K94R | -285.84 | E96H_K94R | -296.03 |
| L43F | -260.04 | N7S | -265.36 | L43F_N7S | -268.35 |
| K19T | -295.79 | T97M | -284.71 | K19T_T97M | -299.55 |
| Q35L | -249.13 | V36M | -259.49 | Q35L_V36M | -275.14 |
| Q54H | -274.04 | R57T | -262.19 | Q54H_R57T | -293.85 |
| N7H | -283.52 | S61T | -282.37 | N7H_S61T | -290.68 |
| Y32W | -268.94 | K24G | -301.98 | Y32W_K24G | -304.19 |
| S46F | -265.46 | G83D | -280.55 | S46F_G83D | -286.33 |
| P10A | -245.53 | S46F | -265.46 | P10A_S46F | -270.89 |
| D98E | -296.01 | P99T | -307.46 | D98E_P99T | -312.58 |
| G42T | -277.23 | T40E | -286.57 | G42T_T40E | -307.42 |
| Y47H | -268.76 | K29Q | -271.81 | Y47H_K29Q | -283.12 |
| Q54H | -274.04 | Y26H | -255.26 | Q54H_Y26H | -289.56 |
| C30Y | -256.53 | K19E | -268.13 | C30Y_K19E | -283.17 |
| H13P | -257.89 | K71Q | -274.87 | H13P_K71Q | -286.55 |
| P68S | -276.48 | L74R | -248.76 | P68S_L74R | -284.07 |
| Q35P | -268.22 | T82A | -268.92 | Q35P_T82A | -276.55 |
| R53K | -305.75 | Y47N | -280.67 | R53K_Y47N | -312.86 |
| G83T | -283.31 | E96T | -287.57 | G83T_E96T | -302.57 |
| L69K | -267.57 | T40N | -274.25 | L69K_T40N | -291.55 |
| Y26H | -255.26 | E92A | -280.46 | Y26H_E92A | -294.02 |
| S62G | -278.94 | K29V | -279.88 | S62G_K29V | -286.85 |
| N67A | -281.08 | S61R | -270.35 | N67A_S61R | -285.7 |
| Q54H | -274.04 | R78H | -271 | Q54H_R78H | -288.61 |
| C25S | -273.76 | C30R | -251.63 | C25S_C30R | -274.3 |
| N7H | -283.52 | P68I | -269.54 | N7H_P68I | -290.7 |
| T77P | -258.91 | P3V | -298.11 | T77P_P3V | -305.02 |
| S93R | -282.4 | T40H | -292.12 | S93R_T40H | -297.49 |
| S62R | -260.63 | E96T | -287.57 | S62R_E96T | -300.05 |
| C27S | -269.5 | K19E | -268.13 | C27S_K19E | -272.16 |
| T77A | -264.63 | A21D | -276.44 | T77A_A21D | -300.92 |
| S87E | -286.86 | E96T | -287.57 | S87E_E96T | -287.68 |
| Q54H | -274.04 | D101T | -265.09 | Q54H_D101T | -279.73 |
| Q54H | -274.04 | Y47N | -280.67 | Q54H_Y47N | -285.27 |
| K71N | -287.4 | N67D | -273.89 | K71N_N67D | -299.03 |
| R49S | -281.74 | N7K | -270.87 | R49S_N7K | -287.81 |
| R57S | -281.31 | I45L | -259.51 | R57S_I45L | -281.74 |
| E92A | -280.46 | D101T | -265.09 | E92A_D101T | -298.76 |
| K90E | -267.44 | K29C | -270.92 | K90E_K29C | -297.16 |
| Q76I | -285.79 | R57K | -272.68 | Q76I_R57K | -300.04 |
| V36L | -279.29 | F100Q | -290.1 | V36L_F100Q | -296.05 |
| Y32W | -268.94 | Q35V | -267.8 | Y32W_Q35V | -285.92 |
| E92A | -280.46 | Y47N | -280.67 | E92A_Y47N | -284.81 |
| P68L | -289.73 | S61H | -272.32 | P68L_S61H | -293.13 |
| K24E | -300.5 | N7K | -270.87 | K24E_N7K | -303.55 |
| C31S | -297.46 | D64V | -283.54 | C31S_D64V | -297.56 |
| A21N | -262.8 | K29R | -288.91 | A21N_K29R | -291.7 |
| S46I | -290.12 | K88E | -302.64 | S46I_K88E | -302.94 |
| D80I | -273.04 | K29Q | -271.81 | D80I_K29Q | -278.26 |
| P81H | -276.78 | E96T | -287.57 | P81H_E96T | -311.97 |
| D80N | -290.84 | D98T | -284.92 | D80N_D98T | -292.28 |
| L39T | -289.47 | H13Q | -268.62 | L39T_H13Q | -290.32 |
| R49S | -281.74 | L74S | -262.04 | R49S_L74S | -309.92 |
| S87E | -286.86 | E86K | -276.45 | S87E_E86K | -296.81 |
| P6F | -275.25 | L39V | -274.16 | P6F_L39V | -277.73 |
| Y32F | -290.79 | P3Q | -297 | Y32F_P3Q | -301.26 |
| P6F | -275.25 | Q60E | -281.96 | P6F_Q60E | -291.32 |
| T40A | -283.29 | N7R | -271.97 | T40A_N7R | -287.15 |
| S61G | -263.22 | P68F | -248.45 | S61G_P68F | -293.66 |
| S93R | -282.4 | K29L | -269.29 | S93R_K29L | -286.56 |
| L39I | -275.65 | Q35V | -267.8 | L39I_Q35V | -293.28 |
| C31R | -264.52 | L8I | -272.01 | C31R_L8I | -280.19 |
| N7R | -271.97 | K29F | -282.27 | N7R_K29F | -287.5 |
| P6F | -275.25 | K24R | -265.04 | P6F_K24R | -286.72 |
| H33P | -257.9 | K71E | -283.65 | H33P_K71E | -304.81 |
| A21H | -278.03 | E9D | -275.49 | A21H_E9D | -303.89 |
| Y47H | -268.76 | S46Y | -261.06 | Y47H_S46Y | -279.91 |
| V36L | -279.29 | K29C | -270.92 | V36L_K29C | -280.75 |
| P59H | -273.74 | K24R | -265.04 | P59H_K24R | -287.23 |
| P68D | -271.06 | N67D | -273.89 | P68D_N67D | -299.85 |
| D80F | -268.1 | P73S | -266.54 | D80F_P73S | -286.66 |
| E9D | -275.49 | R78G | -267.8 | E9D_R78G | -276.74 |
| P68Y | -292.56 | D101A | -279.24 | P68Y_D101A | -302.34 |
| Y47F | -270.1 | S46F | -265.46 | Y47F_S46F | -303.91 |
| G42A | -276.33 | K24Q | -266.1 | G42A_K24Q | -297.97 |
| F38S | -267.09 | S61G | -263.22 | F38S_S61G | -276.86 |
| K29I | -294.62 | H65D | -267.41 | K29I_H65D | -299.17 |
| P84E | -284.55 | K90E | -267.44 | P84E_K90E | -291.44 |
| K24G | -301.98 | D101A | -279.24 | K24G_D101A | -302.64 |
| K24T | -268.14 | A21D | -276.44 | K24T_A21D | -283.99 |
| R56H | -287.75 | P73S | -266.54 | R56H_P73S | -287.95 |
| T77P | -258.91 | P3Q | -297 | T77P_P3Q | -311.22 |
| T77I | -289.79 | T40S | -282.79 | T77I_T40S | -295.05 |
| V91K | -297.51 | Q60R | -293.72 | V91K_Q60R | -298.35 |
| Q63E | -264.72 | P59A | -269.17 | Q63E_P59A | -285.78 |
| S62R | -260.63 | S70Q | -281.04 | S62R_S70Q | -289.11 |
| D80I | -273.04 | K19E | -268.13 | D80I_K19E | -285.15 |
| T77P | -258.91 | R56P | -281.85 | T77P_R56P | -298.23 |
| S62N | -295.25 | P6H | -278.82 | S62N_P6H | -311.49 |
| Q60E | -281.96 | H65N | -273.03 | Q60E_H65N | -282.8 |
| P59H | -273.74 | Q60E | -281.96 | P59H_Q60E | -285.49 |
| N67A | -281.08 | H13Q | -268.62 | N67A_H13Q | -299.56 |
| P59H | -273.74 | Q60S | -268.95 | P59H_Q60S | -307.02 |
| E96K | -296.76 | K94T | -274.84 | E96K_K94T | -300.3 |
| P84S | -280.88 | P59D | -285.21 | P84S_P59D | -287.05 |
| N7R | -271.97 | L69T | -265.57 | N7R_L69T | -286.71 |
| S61G | -263.22 | K29C | -270.92 | S61G_K29C | -296.3 |
| Q76I | -285.79 | T97V | -299.7 | Q76I_T97V | -300.33 |
| L69I | -280.22 | T95R | -279 | L69I_T95R | -286.42 |
| N67A | -281.08 | P3I | -281.45 | N67A_P3I | -301.38 |
| D80N | -290.84 | Y32M | -268.91 | D80N_Y32M | -290.93 |
| S87E | -286.86 | T40N | -274.25 | S87E_T40N | -299.91 |
| T77A | -264.63 | A21N | -262.8 | T77A_A21N | -300.04 |
| E2D | -279.84 | P3Q | -297 | E2D_P3Q | -309.45 |
| L74S | -262.04 | A21N | -262.8 | L74S_A21N | -301.67 |
| Q63E | -264.72 | K94A | -287.25 | Q63E_K94A | -290.62 |
| Y32W | -268.94 | T40A | -283.29 | Y32W_T40A | -304.82 |
| E9D | -275.49 | L74P | -254.07 | E9D_L74P | -294.25 |
| K19A | -278.15 | S93K | -294.38 | K19A_S93K | -304.83 |
| P6F | -275.25 | S87Q | -287.05 | P6F_S87Q | -292.72 |
| P6F | -275.25 | D64A | -282.28 | P6F_D64A | -285.04 |
| Q63E | -264.72 | Y32M | -268.91 | Q63E_Y32M | -297.04 |
| D98H | -289.62 | S61R | -270.35 | D98H_S61R | -303.33 |
| N12K | -276.05 | P6L | -273.65 | N12K_P6L | -291.12 |
| T95A | -283.09 | S46C | -263.06 | T95A_S46C | -290.01 |
| L74F | -293.37 | S61N | -293.52 | L74F_S61N | -300.94 |
| L74A | -290.96 | N7D | -284.34 | L74A_N7D | -303.28 |
| Q54H | -274.04 | N7D | -284.34 | Q54H_N7D | -296.9 |
| P84S | -280.88 | T58N | -263.28 | P84S_T58N | -308.27 |
| N67V | -284.14 | D98T | -284.92 | N67V_D98T | -289.34 |
| E92A | -280.46 | N7D | -284.34 | E92A_N7D | -285.29 |
| D98H | -289.62 | D64G | -263.52 | D98H_D64G | -298.93 |
| Q76I | -285.79 | P84T | -302.63 | Q76I_P84T | -305.67 |
| T82N | -274.68 | Y32F | -290.79 | T82N_Y32F | -294.5 |
| L39T | -289.47 | L74R | -248.76 | L39T_L74R | -300.54 |
| N7R | -271.97 | Q76V | -253.73 | N7R_Q76V | -304.35 |
| S87Q | -287.05 | H65Y | -268.01 | S87Q_H65Y | -298.04 |
| H65R | -262.66 | T97P | -291.5 | H65R_T97P | -297.93 |
| N12T | -275.44 | D101A | -279.24 | N12T_D101A | -281.46 |
| T58S | -242.39 | R56H | -287.75 | T58S_R56H | -289.79 |
| N67V | -284.14 | P68N | -274.71 | N67V_P68N | -299.01 |
| Y32W | -268.94 | Q35M | -266.26 | Y32W_Q35M | -269.39 |
| S62D | -265.66 | S93T | -299.39 | S62D_S93T | -307.71 |
| N67G | -281.99 | T82A | -268.92 | N67G_T82A | -301.12 |
| S61R | -270.35 | H65N | -273.03 | S61R_H65N | -277.41 |
| K24Q | -266.1 | K29Q | -271.81 | K24Q_K29Q | -299.5 |
| Q54H | -274.04 | P59D | -285.21 | Q54H_P59D | -292.42 |
| K29H | -271.18 | S62C | -263.74 | K29H_S62C | -301.47 |
| K71E | -283.65 | K29C | -270.92 | K71E_K29C | -299.11 |
| L74A | -290.96 | Q76N | -278.36 | L74A_Q76N | -303.66 |
| N12T | -275.44 | Q54H | -274.04 | N12T_Q54H | -305.5 |
| L74A | -290.96 | D98T | -284.92 | L74A_D98T | -307.13 |
| T77L | -265.21 | D80E | -289.54 | T77L_D80E | -301.3 |
| N12K | -276.05 | L69E | -251.54 | N12K_L69E | -279.34 |
| Y32W | -268.94 | S46C | -263.06 | Y32W_S46C | -292.81 |
| Q63K | -251.32 | G79E | -239.49 | Q63K_G79E | -287.11 |
| R53I | -284.11 | Y32W | -268.94 | R53I_Y32W | -289.27 |
| F100E | -274.32 | K29V | -279.88 | F100E_K29V | -286.11 |
| D80V | -291.82 | R53G | -296.64 | D80V_R53G | -299.82 |
| S70Q | -281.04 | Q63P | -262.37 | S70Q_Q63P | -292.03 |
| N12K | -276.05 | P3M | -286.02 | N12K_P3M | -294.41 |
| L74T | -287.29 | K19I | -277.11 | L74T_K19I | -289.06 |
| N12E | -276.28 | E86K | -276.45 | N12E_E86K | -292.68 |
| P84S | -280.88 | K94A | -287.25 | P84S_K94A | -292.19 |
| S62H | -262.59 | P59H | -273.74 | S62H_P59H | -306.39 |
| T20A | -268.29 | Q63K | -251.32 | T20A_Q63K | -291.64 |
| T77P | -258.91 | Q76V | -253.73 | T77P_Q76V | -299.26 |
| D80I | -273.04 | K19Q | -273.72 | D80I_K19Q | -298.03 |
| T82A | -268.92 | Q60R | -293.72 | T82A_Q60R | -298.01 |
| N23T | -284.64 | A21G | -275.66 | N23T_A21G | -307.31 |
| H65R | -262.66 | K29Q | -271.81 | H65R_K29Q | -304.03 |
| L39I | -275.65 | Q35M | -266.26 | L39I_Q35M | -276.2 |
| V4I | -266.35 | F100K | -281.48 | V4I_F100K | -285.09 |
| L39I | -275.65 | L69T | -265.57 | L39I_L69T | -281.21 |
| S93R | -282.4 | Q76V | -253.73 | S93R_Q76V | -284.31 |
| N7K | -270.87 | L39A | -290.22 | N7K_L39A | -306.02 |
| T77I | -289.79 | T97S | -280.71 | T77I_T97S | -291.7 |
| V36I | -280.01 | K19Q | -273.72 | V36I_K19Q | -291.06 |
| R56P | -281.85 | Q54H | -274.04 | R56P_Q54H | -283.64 |
| L39N | -267.77 | Q35L | -249.13 | L39N_Q35L | -281.85 |
| R53S | -281.99 | Q17R | -279.94 | R53S_Q17R | -287.21 |
| Q63E | -264.72 | T58D | -266.52 | Q63E_T58D | -270.93 |
| S70P | -241.61 | F100P | -257.18 | S70P_F100P | -278.75 |
| F100R | -262.78 | Q60R | -293.72 | F100R_Q60R | -305.13 |
| N67V | -284.14 | L69T | -265.57 | N67V_L69T | -305.51 |
| E92T | -268.91 | K90E | -267.44 | E92T_K90E | -288.49 |
| N7R | -271.97 | N12S | -276.68 | N7R_N12S | -282.29 |
| D98H | -289.62 | T97S | -280.71 | D98H_T97S | -296 |
| N67D | -273.89 | E96R | -270.13 | N67D_E96R | -274.09 |
| G15V | -265.74 | N7S | -265.36 | G15V_N7S | -287.26 |
| Q60P | -264.92 | T58D | -266.52 | Q60P_T58D | -277.57 |
| N7R | -271.97 | R78N | -270.99 | N7R_R78N | -292.28 |
| K19E | -268.13 | K24Q | -266.1 | K19E_K24Q | -272.55 |
| E86K | -276.45 | K71N | -287.4 | E86K_K71N | -294.75 |
| L74A | -290.96 | Q76L | -272.77 | L74A_Q76L | -294.73 |
| R53K | -305.75 | S46C | -263.06 | R53K_S46C | -308.14 |
| Y26F | -285.42 | K94T | -274.84 | Y26F_K94T | -294 |
| E96R | -270.13 | P68L | -289.73 | E96R_P68L | -292.06 |
| G83D | -280.55 | L69P | -262.23 | G83D_L69P | -287.38 |
| T77P | -258.91 | K94R | -285.84 | T77P_K94R | -294.75 |
| P84S | -280.88 | P59Y | -308.07 | P84S_P59Y | -308.16 |
| K89E | -297.76 | T97R | -305.65 | K89E_T97R | -307.13 |
| T97P | -291.5 | S70R | -265.4 | T97P_S70R | -302.03 |
| K29R | -288.91 | Q76L | -272.77 | K29R_Q76L | -293.06 |
| K51E | -297.44 | K85E | -306.4 | K51E_K85E | -311.37 |
| L74F | -293.37 | Q17R | -279.94 | L74F_Q17R | -299.78 |
| C31S | -297.46 | Q54S | -306.35 | C31S_Q54S | -307.61 |
| S93T | -299.39 | L74F | -293.37 | S93T_L74F | -308.79 |
| T77P | -258.91 | R78N | -270.99 | T77P_R78N | -297.99 |
| Q54N | -275.61 | E2D | -279.84 | Q54N_E2D | -280.73 |
| P6H | -278.82 | Y47H | -268.76 | P6H_Y47H | -284.61 |
| P59S | -276.01 | Q54K | -262.05 | P59S_Q54K | -305.03 |
| D80V | -291.82 | S62C | -263.74 | D80V_S62C | -297.12 |
| S62I | -281.16 | L69I | -280.22 | S62I_L69I | -291.21 |
| S93R | -282.4 | G79W | -273.59 | S93R_G79W | -295.13 |
| Q76H | -302.88 | K29A | -297.02 | Q76H_K29A | -305.64 |
| P84Q | -266.4 | P68F | -248.45 | P84Q_P68F | -294.1 |
| E2D | -279.84 | K94R | -285.84 | E2D_K94R | -294.37 |
| S61T | -282.37 | S75P | -295.3 | S61T_S75P | -299.75 |
| S93R | -282.4 | R78N | -270.99 | S93R_R78N | -290.4 |
| T97K | -266.75 | T77L | -265.21 | T97K_T77L | -288.81 |
| A21D | -276.44 | T82A | -268.92 | A21D_T82A | -291.53 |
| L39S | -290.98 | T40N | -274.25 | L39S_T40N | -303.26 |
| P68S | -276.48 | K90A | -271.68 | P68S_K90A | -300.56 |
| S75P | -295.3 | K71G | -297.66 | S75P_K71G | -305.06 |
| Q35P | -268.22 | N12R | -275.1 | Q35P_N12R | -285.46 |
| S61N | -293.52 | Q60L | -252.74 | S61N_Q60L | -294.44 |
| K29P | -270.26 | S93R | -282.4 | K29P_S93R | -286.05 |
| S70P | -241.61 | K19V | -263.09 | S70P_K19V | -281.54 |
| D64A | -282.28 | T97K | -266.75 | D64A_T97K | -304.59 |
| S93G | -300.22 | N67T | -304.28 | S93G_N67T | -307.16 |
| R78Q | -269.18 | D101L | -307.18 | R78Q_D101L | -307.65 |
| P59H | -273.74 | K94T | -274.84 | P59H_K94T | -311.77 |
| N12K | -276.05 | R53Q | -254.76 | N12K_R53Q | -288.41 |
| E2D | -279.84 | Q76E | -279.36 | E2D_Q76E | -283.21 |
| Y32W | -268.94 | Q76E | -279.36 | Y32W_Q76E | -298.86 |
| E92A | -280.46 | L74D | -275.08 | E92A_L74D | -285.03 |
| E96G | -271.66 | D64A | -282.28 | E96G_D64A | -290.99 |
| T95A | -283.09 | E96V | -274.37 | T95A_E96V | -305.89 |
| N12E | -276.28 | T82A | -268.92 | N12E_T82A | -282.06 |
| E2D | -279.84 | A21V | -259.47 | E2D_A21V | -289.31 |
| T77P | -258.91 | K90A | -271.68 | T77P_K90A | -279.22 |
| P59T | -277.96 | E9D | -275.49 | P59T_E9D | -295.68 |
| Q63K | -251.32 | P59R | -252.07 | Q63K_P59R | -278.66 |
| T97E | -276.06 | K71E | -283.65 | T97E_K71E | -298.81 |
| R52W | -292.52 | R53S | -281.99 | R52W_R53S | -312.78 |
| F100Y | -300.91 | K88T | -286.72 | F100Y_K88T | -302.57 |
| Q63K | -251.32 | L69K | -267.57 | Q63K_L69K | -302.5 |
| S93K | -294.38 | T77Q | -293.61 | S93K_T77Q | -307.99 |
| Q60P | -264.92 | E92K | -265.06 | Q60P_E92K | -267.04 |
| T77A | -264.63 | S61E | -296.98 | T77A_S61E | -298.03 |
| N7R | -271.97 | R55G | -272.14 | N7R_R55G | -277.13 |
| T97S | -280.71 | K19E | -268.13 | T97S_K19E | -291.77 |
| K71Q | -274.87 | K19A | -278.15 | K71Q_K19A | -278.35 |
| S93R | -282.4 | E92K | -265.06 | S93R_E92K | -307.33 |
| E2D | -279.84 | S87K | -271.11 | E2D_S87K | -295.92 |
| N67D | -273.89 | L8Q | -278.41 | N67D_L8Q | -305.63 |
| T97D | -275.9 | Q63E | -264.72 | T97D_Q63E | -287.38 |
| K94S | -276.34 | D101C | -262.42 | K94S_D101C | -278.47 |
| E96R | -270.13 | T77L | -265.21 | E96R_T77L | -302.07 |
| N12R | -275.1 | F100R | -262.78 | N12R_F100R | -277.49 |
| F100Y | -300.91 | D98A | -281.03 | F100Y_D98A | -305.44 |
| D80P | -270.23 | T97I | -273.94 | D80P_T97I | -306.93 |
| F100R | -262.78 | L69P | -262.23 | F100R_L69P | -268.47 |
| D80N | -290.84 | T95E | -297.01 | D80N_T95E | -306.82 |
| E2D | -279.84 | S46V | -261.47 | E2D_S46V | -294.94 |
| T58N | -263.28 | P84Q | -266.4 | T58N_P84Q | -280.45 |
| P84Q | -266.4 | K29F | -282.27 | P84Q_K29F | -284.34 |
| T58N | -263.28 | E86K | -276.45 | T58N_E86K | -296.64 |
| K94R | -285.84 | K71E | -283.65 | K94R_K71E | -304.98 |
| T95E | -297.01 | Q60P | -264.92 | T95E_Q60P | -299.66 |
| R49S | -281.74 | N7R | -271.97 | R49S_N7R | -294.4 |
| S16C | -281.41 | S61G | -263.22 | S16C_S61G | -297.51 |
| R53I | -284.11 | A21P | -274.64 | R53I_A21P | -307.52 |
| V91A | -296.91 | R57S | -281.31 | V91A_R57S | -304.81 |
| K29L | -269.29 | Q63P | -262.37 | K29L_Q63P | -286.24 |
| T82K | -292.43 | E96T | -287.57 | T82K_E96T | -308.46 |
| G83V | -285.21 | G79R | -276.99 | G83V_G79R | -290.96 |
| S46C | -263.06 | T58S | -242.39 | S46C_T58S | -267.69 |
| T77N | -264.83 | K29V | -279.88 | T77N_K29V | -302.16 |
| D101V | -233.18 | F100R | -262.78 | D101V_F100R | -294.6 |
| N7H | -283.52 | D80H | -282.31 | N7H_D80H | -293.87 |
| C25S | -273.76 | D80H | -282.31 | C25S_D80H | -293.03 |
| P68N | -274.71 | Q54P | -232.51 | P68N_Q54P | -301.13 |
| K19H | -271.62 | D80H | -282.31 | K19H_D80H | -284.82 |
| Q17R | -279.94 | T77N | -264.83 | Q17R_T77N | -280.22 |
| K90A | -271.68 | Q76V | -253.73 | K90A_Q76V | -287.11 |
| P18L | -267.06 | Q54H | -274.04 | P18L_Q54H | -297.84 |
| P59S | -276.01 | T97N | -289.01 | P59S_T97N | -293.37 |
| S87E | -286.86 | Q66H | -294.8 | S87E_Q66H | -309.94 |
| C22W | -268.13 | S46P | -268.79 | C22W_S46P | -272.55 |
| R56Q | -278.9 | K24R | -265.04 | R56Q_K24R | -279.42 |
| V36G | -280.89 | P68S | -276.48 | V36G_P68S | -303.57 |
| P68S | -276.48 | H65S | -273.2 | P68S_H65S | -276.51 |
| C22W | -268.13 | L8R | -254.58 | C22W_L8R | -270.04 |
| K28N | -269.18 | D80H | -282.31 | K28N_D80H | -285.41 |
| T77C | -270.78 | K90E | -267.44 | T77C_K90E | -290.57 |
| I45F | -264.1 | L8R | -254.58 | I45F_L8R | -272.98 |
| E86G | -269.06 | S61T | -282.37 | E86G_S61T | -295.25 |
| N7K | -270.87 | L8Q | -278.41 | N7K_L8Q | -280.7 |
| N7R | -271.97 | E96I | -292.72 | N7R_E96I | -304.13 |
| E96N | -270.79 | N7R | -271.97 | E96N_N7R | -288.33 |
| S62G | -278.94 | T97E | -276.06 | S62G_T97E | -286.63 |
| Q76L | -272.77 | Q54P | -232.51 | Q76L_Q54P | -301.13 |
| G79K | -288.26 | S93R | -282.4 | G79K_S93R | -298.22 |
| K94S | -276.34 | T58G | -272.44 | K94S_T58G | -293.22 |
| E2D | -279.84 | E86G | -269.06 | E2D_E86G | -283.74 |
| S87E | -286.86 | R57P | -298.12 | S87E_R57P | -301.92 |
| P6S | -292.28 | T82N | -274.68 | P6S_T82N | -299.27 |
| T82N | -274.68 | N67D | -273.89 | T82N_N67D | -276.12 |
| T97D | -275.9 | S61T | -282.37 | T97D_S61T | -287.67 |
| P3I | -281.45 | D101R | -263.2 | P3I_D101R | -285.08 |
| K88S | -298.14 | R57P | -298.12 | K88S_R57P | -307.85 |
| K88A | -288.38 | K19D | -259.76 | K88A_K19D | -294.22 |
| R78N | -270.99 | G44R | -267.15 | R78N_G44R | -283.16 |
| Q17K | -279.19 | A21K | -276.66 | Q17K_A21K | -292.98 |
| D98Y | -270.67 | S61N | -293.52 | D98Y_S61N | -298.67 |
| G15V | -265.74 | N7R | -271.97 | G15V_N7R | -285.01 |
| T95K | -280.22 | T58G | -272.44 | T95K_T58G | -287.91 |
| D80G | -279.14 | Y26F | -285.42 | D80G_Y26F | -298.87 |

**Supplementary Methods:**

**Classification of Compensatory Mutations with Machine learning**

**Sequence One-Hot Encoding and Associated Amino Acid Embedding:**

To prepare input features for the classification model, we applied two levels of encoding: global and association-specific. Full-length HIV-1 Tat protein sequences (101 amino acids) were encoded using a 20-dimensional one-hot representation, where each amino acid (e.g., alanine, valine, leucine, etc.) was mapped to a unique binary vector reflecting its identity across the entire sequence (1). For codon–codon associated mutation pairs, we applied a 5-dimensional physicochemical embedding based on amino acid properties: non-polar, polar uncharged, positively charged (basic), negatively charged (acidic), and special cases (e.g., proline, glycine). This dual encoding strategy allowed the model to capture both global sequence-level patterns and local biochemical context at associated mutation sites, enabling effective classification of compensatory mutations (2).

**Co-occurrences of Amino Acid:**

The presence or absence of specific amino acids at associated position pairs, identified through the phylogenetic dependency network (PDN), was quantified using a 2×2 contingency table framework (TT, TF, FT, FF), representing co-occurrence patterns across codon–codon associations (3).

**Selection Pressure and Amino Acid Variability Per Site**

Site-specific evolutionary patterns were assessed by calculating the dn/ds ratio (selection pressure) and Shannon entropy (amino acid variability) for each position in the Tat protein. The dn/ds ratio reflects the nature of selection—positive, neutral, or purifying—while entropy scores indicate the degree of sequence variability, with higher values representing greater amino acid diversity at a given site.

**Structural Features:**

To evaluate the structural context of mutations, several features were analyzed. Cα–Cα distances between mutation sites were computed using Biopython to assess physical proximity, as spatially adjacent residues are more likely to interact structurally. Solvent Accessible Surface Area (SASA) was calculated using FreeSASA via Biopython, distinguishing buried from exposed residues—buried residues often play a critical role in structural stability (4). Secondary structure assignments were determined using DSSP, as mutations within helices and β-sheets are generally more constrained due to reduced flexibility (5). Additionally, the proximity of mutations to functional motifs—such as the basic (arginine-rich) domain—was assessed, as mutations in these regions are more likely to impact function and potentially require compensatory changes. Finally, the predicted change in folding stability (ΔΔG) for each mutation was estimated using DynaMut2, providing a quantitative measure of how each substitution may destabilize the protein, thereby indicating structural or functional fitness valleys (6).

**Labeling Feature**

Molecular docking was performed using HDOCK to evaluate the binding affinity between HIV-1 Tat variants and TAR RNA (7). The wild-type Tat protein was assumed to possess optimal structural stability and binding affinity. Single mutations that resulted in reduced docking scores (i.e., weaker binding) were interpreted as disruption of native function. Double mutants were evaluated to determine if they partially restored binding compared to single mutants. If a double mutant exhibited stronger binding than both corresponding single mutants but still weaker than the wild type, it was considered a compensatory mutation and labeled as 1. In contrast, if the double mutant did not significantly improve binding over single mutants, it was considered non-compensatory and labeled as 0. Any double mutant that demonstrated stronger binding than the wild type was excluded from the dataset, as such gain-of-function effects are biologically rare in the context of compensatory evolution and may represent artifacts or non-compensatory phenomena.

**Random Oversampling**

To address class imbalance between compensatory (label 1) and non-compensatory (label 0) mutation instances, we applied random oversampling using a custom Python script. The minority class (label 1) was randomly duplicated until both classes were equally represented, ensuring a balanced dataset for downstream classification and minimizing model bias toward the majority class (8, 9).

**Model Architecture:**

A supervised binary classification framework was developed and implemented in Python using the scikit-learn library. Three machine-learning algorithms were evaluated: Random Forest (RF), Support Vector Machine (SVM), and Decision Tree (DT). The training dataset consisted of engineered features extracted from amino acid sequences and codon–codon associations, with feature vectors spanning columns C to CAK and the binary class label (compensatory vs. non-compensatory) in column CAL.

For the Random Forest model, 100 decision trees were used (n_estimators = 100) with a fixed random seed (random_state = 42) to ensure reproducibility. The Decision Tree classifier was implemented using default scikit-learn parameters with controlled random initialization for consistency. The Support Vector Machine classifier was trained using a radial basis function (RBF) kernel, with hyperparameters selected using validation performance.

Model performance was evaluated using three independent datasets: training (70%), validation (10%), and test (20%), each maintaining a consistent feature structure. Accuracy scores were calculated for all models across each dataset. Confusion matrices were generated to quantify true positives, true negatives, false positives, and false negatives, and were visualized using ConfusionMatrixDisplay from sklearn.metrics. Training accuracy was first computed to assess model fitting, while generalization capability was evaluated on the held-out validation set and an entirely unseen test set. This multi-stage evaluation enabled robust comparison of classifier performance on balanced datasets generated through prior random oversampling. All source code, model implementation scripts, and a sample dataset used in this study are available at Data and Code Availability section.

References

1. Bustamam A, Musti MIS, Hartomo S, Aprilia S, Tampubolon PP, Lestari D. Performance of rotation forest ensemble classifier and feature extractor in predicting protein interactions using amino acid sequences. BMC Genomics. 2019;20(9):950.

2. Wu X, Zeng W, Lin F. GCNCPR-ACPs: a novel graph convolution network method for ACPs prediction. BMC bioinformatics. 2022;23(Suppl 4):560.

3. Carlson JM, Brumme ZL, Rousseau CM, Brumme CJ, Matthews P, Kadie C, et al. Phylogenetic dependency networks: inferring patterns of CTL escape and codon covariation in HIV-1 Gag. PLoS computational biology. 2008;4(11):e1000225.

4. Mitternacht S. FreeSASA: An open source C library for solvent accessible surface area calculations [version 1; peer review: 2 approved]. F1000Research. 2016;5(189).

5. Kabsch W, Sander C. Dictionary of protein secondary structure: pattern recognition of hydrogen-bonded and geometrical features. Biopolymers. 1983;22(12):2577-637.

6. Rodrigues CHM, Pires DEV, Ascher DB. DynaMut2: Assessing changes in stability and flexibility upon single and multiple point missense mutations. Protein science : a publication of the Protein Society. 2021;30(1):60-9.

7. Yan Y, Zhang D, Zhou P, Li B, Huang SY. HDOCK: a web server for protein-protein and protein-DNA/RNA docking based on a hybrid strategy. Nucleic acids research. 2017;45(W1):W365-w73.

9. Lemaître G, Nogueira F, Aridas CK. Imbalanced-learn: a python toolbox to tackle the curse of imbalanced datasets in machine learning. J Mach Learn Res. 2017;18(1):559–63.

8. .
